# CD8^+^ T cell–derived IL-13 increases macrophage IL-10 to resolve neuropathic pain

**DOI:** 10.1172/jci.insight.154194

**Published:** 2022-03-08

**Authors:** Susmita K. Singh, Karen Krukowski, Geoffroy O. Laumet, Drew Weis, Jenolyn F. Alexander, Cobi J. Heijnen, Annemieke Kavelaars

**Affiliations:** 1 Laboratories of Neuroimmunology, Department of Symptom Research, the University of Texas MD Anderson Cancer Center, Houston, Texas, USA.; 2 Department of Biological Sciences, Knoebel Institute for Healthy Aging, University of Denver, Denver, Colorado, USA.; 3 Department of Physiology, College of Natural Science, Michigan State University, East Lansing, Michigan, USA.

**Keywords:** Neuroscience, Cellular immune response, Pain, T cells

## Abstract

Understanding the endogenous mechanisms regulating resolution of pain may identify novel targets for treatment of chronic pain. Resolution of chemotherapy-induced peripheral neuropathy (CIPN) after treatment completion depends on CD8^+^ T cells and on IL-10 produced by other cells. Using *Rag2*^–/–^ mice lacking T and B cells and adoptive transfer of *Il13*^–/–^ CD8^+^ T cells, we showed that CD8^+^ T cells producing IL-13 were required for resolution of CIPN. Intrathecal administration of anti–IL-13 delayed resolution of CIPN and reduced IL-10 production by dorsal root ganglion macrophages. Depleting local CD206^+^ macrophages also delayed resolution of CIPN. In vitro, TIM3^+^CD8^+^ T cells cultured with cisplatin, apoptotic cells, or phosphatidylserine liposomes produced IL-13, which induced IL-10 in macrophages. In vivo, resolution of CIPN was delayed by intrathecal administration of anti-TIM3. Resolution was also delayed in *Rag2*^–/–^ mice reconstituted with *Havcr2* (TIM3)^–/–^ CD8^+^ T cells. Our data indicated that cell damage induced by cisplatin activated TIM3 on CD8^+^ T cells, leading to increased IL-13 production, which in turn induced macrophage IL-10 production and resolution of CIPN. Development of exogenous activators of the IL-13/IL-10 pain resolution pathway may provide a way to treat the underlying cause of chronic pain.

## Graphical abstract

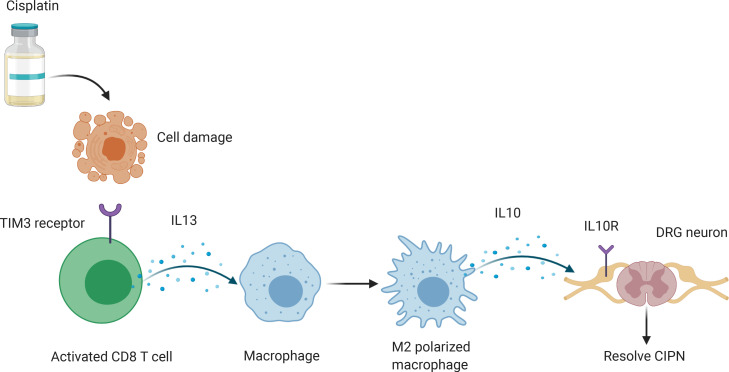

## Introduction

Chronic pain affects between 30% and 50% of the population worldwide and has a profound impact on individuals and society. Acute pain in response to tissue damage or infection serves an adaptive purpose and is usually manageable. When pain persists after the noxious stimulus is removed or the tissue damage or pathology has healed (1–4) and transition to chronic pain occurs, pain control becomes much more challenging. Transition to chronic pain can occur in many different conditions and is frequently reported after surgery, traumatic nerve injury, or after completion of cancer treatment with chemotherapy (5, 6). Patients treated for cancer with chemotherapy often develop peripheral neuropathy characterized by pain and numbness and tingling in the hands and feet. In 70%–75% of patients, chemotherapy-induced peripheral neuropathy (CIPN) resolves within weeks to months after completion of treatment. However, in the other 25%–30% of patients, CIPN persists for more than 3 months to years (7, 8). Understanding the endogenous mechanisms that promote the natural resolution of pain may identify potentially novel targets for the prevention and treatment of chronic pain (2).

It is well known that the immune system plays a critical role in the onset and maintenance of chronic pain (9, 10). Recent findings indicate that the immune system, and in particular T cells, also contributes to pain resolution (2, 10). For example, CD3^+^ T cells play a key role in the resolution of pain induced by complete Freund’s adjuvant (CFA) (11). Tregs identified as CD4^+^FOXP3^+^ T cells can suppress neuropathic pain induced by peripheral nerve ligation and inflammatory pain in a model of neuritis and in models of colitis (11–14). Our previous work showed that CD8^+^ T cells are essential for the resolution of CIPN (15, 16). *Rag2*^–/–^ mice lacking mature T and B cells develop persistent pain after treatment with the chemotherapeutics cisplatin or paclitaxel, and adoptive transfer of CD8^+^ T cells, but not of CD4^+^ T cells, normalizes pain resolution (15, 16). The resolution of CIPN is dependent not only on CD8^+^ T cells but also on endogenous IL-10 signaling to IL-10 receptors expressed on primary sensory neurons. However, CD8^+^ T cells are not the source of the IL-10 needed for resolution (15–17).

IL-13 is a pleiotropic cytokine that can be produced by multiple cells, including CD8^+^ T cells (18). IL-13 signals through a receptor complex consisting of the IL-13–binding IL-13 Rα chain and IL-4 Rα, a subunit shared with the IL-4 receptor. IL-13 receptors are expressed on hematopoietic cells, including macrophages, and on nonhematopoietic cells (19). Triggering of the IL-13 receptor on macrophages can promote IL-10 production (20). IL-13 receptors are also expressed on sensory neurons (21, 22), but the role of endogenous IL-13 in models of chronic pain has not been studied.

We showed previously that CD8^+^ T cells can promote resolution of CIPN only when adoptive transfer of CD8^+^ T cells to *Rag2*^–/–^ mice takes place before treatment with cisplatin (16). Chemotherapeutics like cisplatin induce apoptotic cell death of tumor cells and of healthy dividing cells (23). Therefore, we hypothesize that cisplatin-induced cell damage induces the pain-resolving activity of CD8^+^ T cells. In searching for the underlying pathway, we investigated the contribution of the expression of T cell Ig and mucin domain-containing protein 3 (TIM3) on CD8^+^ T cells. We focused on TIM3 because this receptor can recognize phosphatidylserine (PS) present on the outer leaflet of the cell membrane of apoptotic cells via a PS binding pocket (24). TIM3 is expressed on hematopoietic cells, including CD8^+^ T cells, and was first identified as a marker of T cell exhaustion (25, 26). Recent evidence shows that TIM3 can also contribute to T cell activation (27). Here, we tested the hypothesis that apoptotic cells can induce the pain-resolving activity of CD8^+^ T cells via TIM3 expressed on these cells.

Using adoptive transfer studies, in vitro coculture studies, and flow cytometric analysis, we uncovered a critical role of the cytokine IL-13 produced by TIM3^+^CD8^+^ T cells in resolution of CIPN. IL-13 production by TIM3^+^CD8^+^ T cells was activated by apoptotic cells. We also showed that CD8^+^ T cell–derived IL-13 induced IL-10–producing macrophages during resolution of CIPN and identified a key role of CD206^+^ macrophages in resolution of CIPN.

## Results

### IL-13 production by CD8^+^ T cells is required for resolution of cisplatin-induced mechanical allodynia.

**Figure 1:**
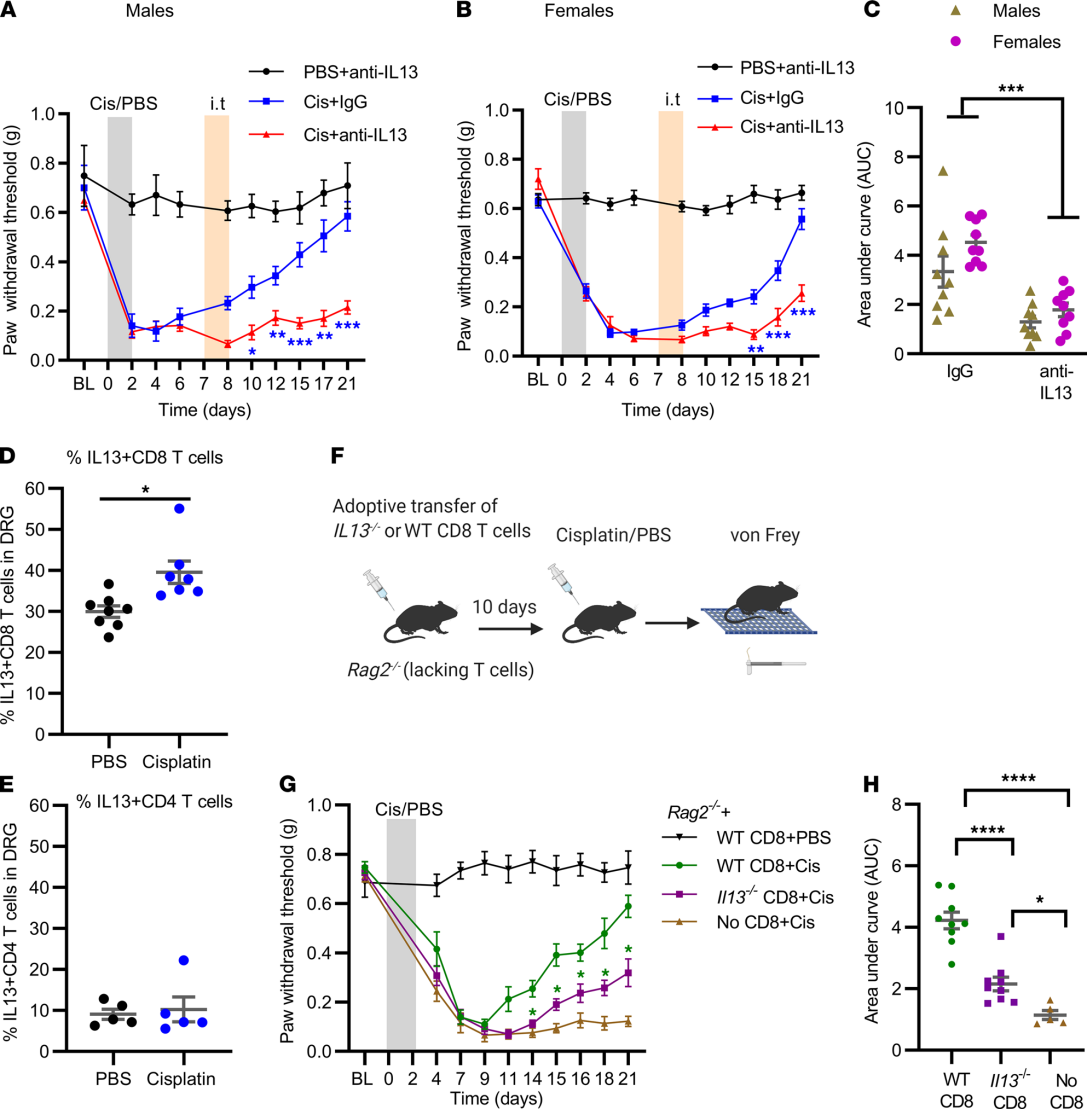
IL-13 production by CD8^+^ T cells is required for resolution of cisplatin-induced mechanical allodynia. (**A**) Male and (**B**) female mice were treated with cisplatin (3 daily doses of 2 mg/kg i.p.); on days 7 and 8, mice were intrathecally injected with anti–IL-13 antibody or control IgG (10 μg/mouse/d) and mechanical allodynia was followed over time. Two-way repeated-measures ANOVA followed by Bonferroni’s multiple-comparison test at individual time points: Cis+IgG versus Cis+anti–IL-13; *P < 0.05, **P < 0.01, ***P < 0.001. Males: PBS+anti–IL-13: *n* = 4; females: Cis+IgG and Cis+anti–IL-13: *n* = 9. (**C**) Resolution of mechanical allodynia expressed as AUC between days 7 and 21. Two-way ANOVA: ***P < 0.001 main effect anti–IL-13; interaction: not significant; Bonferroni’s multiple-comparison: no differences between males and females. (**D**) IL-13^+^CD8^+^ T cells and (**E**) IL-13^+^CD4^+^ T cells in the DRG during CIPN resolution quantified by flow cytometry. Welch’s *t* test; *P < 0.05; *n* = 5 males + 2–3 females/group for **D** and *n* = 5 males/group for **E**. (**F**) Experimental setup for examining the role of IL-13–producing CD8^+^ T cells in resolution of CIPN. (**G**) Time course of change in mechanical sensitivity in male and female *Rag2*^–/–^ mice reconstituted with WT or *Il13*^–/–^ CD8^+^ T cells or not receiving any cells. Two-way repeated-measures ANOVA followed by Bonferroni’s multiple-comparison test at each time point for *Rag2*^–/–^ reconstituted with WT versus *Il13*^–/–^ CD8^+^ T cells: *P < 0.05; *Rag2*^–/–^+WT CD8+PBS, *n* = 3 male, 2 female; *Rag2*^–/–^+WT CD8+Cis, *n* = 5 male, 4 female; *Rag2*^–/–^+*Il13*^–/–^ CD8+Cis, *n* = 5 male, 4 female; *Rag2*^–/–^+No CD8+Cis, *n* = 3 male, 2 female. (**H**) Resolution of mechanical allodynia expressed as AUC between days 11 and 21. One-way ANOVA followed by Tukey’s multiple-comparison test: *P < 0.05; ****P < 0.0001. All data represent mean ± SEM.

Resolution of chemotherapy-induced mechanical allodynia is dependent on CD8^+^ T cells and IL-10, but CD8^+^ T cells are not the source of the IL-10 (15). To determine how CD8^+^ T cells promote production of IL-10 and resolution of CIPN, we investigated the role of IL-13, a cytokine produced by CD8^+^ T cells (16). We selected IL-13 because of evidence that in vitro, IL-13 promotes IL-10 production by macrophages (20). To monitor resolution of CIPN, we assessed mechanical allodynia, the situation in which a normally non-noxious stimulus becomes noxious, using von Frey filaments. Mechanical allodynia, expressed as the force that has a 50% likelihood of inducing a withdrawal response, was used as the readout because it can reliably be monitored over time and is a widely used indicator of the neuronal sensitization that is associated with chronic pain (28). Male and female WT mice were treated with cisplatin (2 mg/kg/d i.p. for 3 days) followed by intrathecal administration of anti–IL-13 or control IgG on days 7 and 8 after the first dose of cisplatin. Intrathecal anti–IL-13 antibody significantly delayed resolution of cisplatin-induced mechanical allodynia in male and female mice (Figure 1, A–C). We did not detect changes in mechanical sensitivity after intrathecal administration of anti–IL-13 to PBS-treated mice (Figure 1, A and B, and Supplemental Figure 1; supplemental material available online with this article; https://doi.org/10.1172/jci.insight.154194DS1). To assess potential sex differences in the effect of anti–IL-13 on resolution of cisplatin-induced mechanical allodynia, we compared the AUC of the resolution phase (Figure 1C). Two-way ANOVA revealed a main effect of anti–IL-13 (*P* < 0.001) but no significant interaction between sex and anti–IL-13, indicating that the effect of anti–IL-13 on resolution did not differ between males and females. Therefore, we pooled data from males and females in subsequent experiments.

Flow cytometric analysis revealed that resolution of CIPN was associated with an increase in IL-13–producing CD8^+^ T cells in the dorsal root ganglia (DRG) (Figure 1D; gating strategy shown in Supplemental Figure 2 and flow plots in Supplemental Figure 3A). We did not detect changes in IL-13–producing CD4^+^ T cells (Figure 1E and Supplemental Figure 3B) or F4/80^+^ macrophages in the DRG of mice treated with cisplatin (Supplemental Figure 3, C and D).

To determine whether CD8^+^ T cells promote resolution of CIPN via production of IL-13, we reconstituted male and female *Rag2*^–/–^ mice that lack mature T cells or B cells with either WT CD8^+^ T cells or with CD8^+^ T cells isolated from the spleen of *Il13*^–/–^ mice. Ten days later, mice were treated with 3 daily doses of cisplatin (2 mg/kg; Figure 1F). Consistent with our previous findings (15, 16), mechanical allodynia did not resolve in *Rag2*^–/–^ mice and reconstitution with WT CD8^+^ T cells restored resolution (Figure 1, G and H). Resolution of cisplatin-induced allodynia was significantly delayed in *Rag2*^–/–^ mice reconstituted with *Il13*^–/–^ CD8^+^ T cells as compared with WT CD8^+^ T cells (Figure 1, G and H). These findings indicated that CD8^+^ T cells capable of producing IL-13 were required for normal resolution of mechanical allodynia induced by cisplatin but that there was also a smaller IL-13–independent contribution of CD8^+^ T cells to resolution. There was no effect of reconstitution of *Rag2*^–/–^ with WT or *Il13*^–/–^ CD8^+^ T cells on mechanical sensitivity at baseline (Figure 1G; 1-way ANOVA of baseline sensitivity: NS). In addition, there were no group differences in maximal allodynia in the cisplatin-treated groups as measured on day 9 (Figure 1G; 1-way ANOVA: NS). The percentage of CD8^+^ T cells in the spleen and DRG of *Rag2*^–/–^ mice was similar after transfer of WT and *Il13*^–/–^ CD8^+^ T cells, indicating that there were no genotype-related differences in reconstitution (Supplemental Figure 4).

### IL-13 promotes IL-10 production by DRG macrophages.

**Figure 2:**
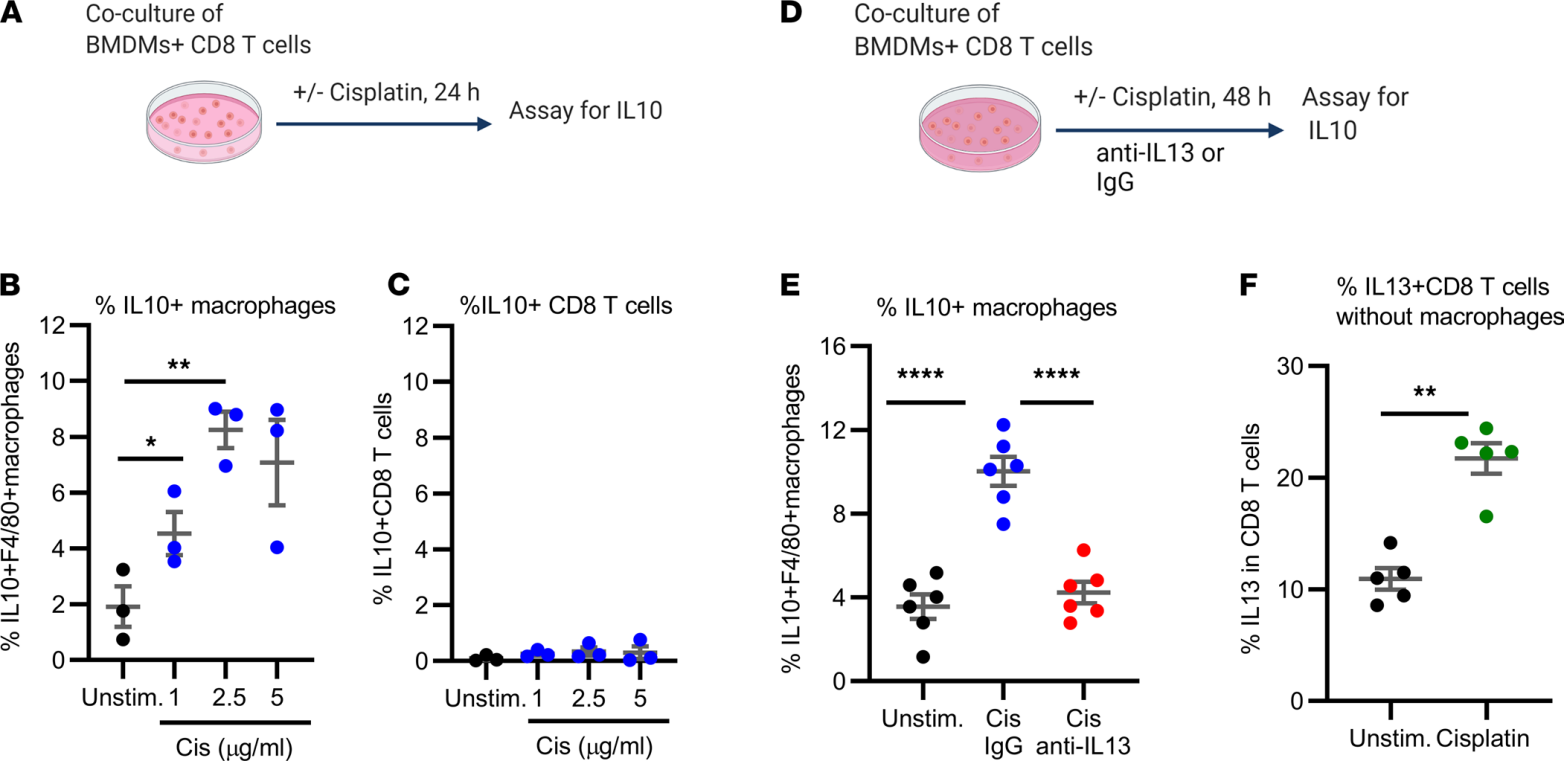
IL-13 mediates the cisplatin-induced increase in IL-10 production by macrophages. (**A**) Schematic representation of cocultures of bone marrow–derived macrophages (BMDMs) and CD8^+^ T cells isolated from spleen treated in vitro with cisplatin (1, 2.5, and 5 μg/mL) or left untreated (unstim.); 24 hours later, the cells were assayed for (**B**) IL-10–containing F4/80^+^ macrophages and (**C**) CD8^+^ T cells by flow cytometry. One-way ANOVA followed by Dunnett’s multiple-comparison test, *P < 0.05, **P < 0.01; *n* = 3 independent experiments; 2 male, 1 female. (**D**) Schematic representation of cocultures of BMDMs and CD8^+^ T cells treated with cisplatin (1 μg/mL) in the presence of neutralizing anti–IL-13 antibody or control IgG or left untreated (unstim.). (**E**) Cells were assayed for IL-10^+^F4/80^+^macrophages by flow cytometry 48 hours later. One-way ANOVA followed by Tukey’s multiple-comparison test. ****P < 0.0001; *n* = 6 independent experiments; 3 male, 3 female. (**F**) Isolated CD8^+^ T cells were cultured with or without cisplatin (1 μg/mL) and assayed for IL-13 by flow cytometry. Welch’s *t* test; **P < 0.01; *n* = 5 independent experiments; 2 male, 3 female. All data are shown as mean ± SEM.

Resolution of CIPN depends on IL-10 signaling to IL-10 receptors expressed on DRG neurons (17), but the source of the IL-10 is not known. To identify the potential source of IL-10, we performed in vitro cocultures of bone marrow–derived macrophages (BMDMs) with CD8^+^ T cells isolated from the spleen (Figure 2A). Stimulation of these cocultures with cisplatin dose dependently increased the production of IL-10 by macrophages (Figure 2, A and B, and Supplemental Figure 5A). We did not detect induction of IL-10 in CD8^+^ T cells (Figure 2, A and C, and Supplemental Figure 5B), which is consistent with our previous in vivo data showing that CD8^+^ T cells are not the source of the IL-10 needed for resolution of CIPN (15). Addition of anti–IL-13 to cocultures of CD8^+^ T cells and macrophages stimulated with cisplatin prevented the increase in macrophage IL-10 production (Figure 2, D and E, and Supplemental Figure 5C). Cisplatin did not increase macrophage IL-10 production in cultures without CD8^+^ T cells (Supplemental Figure 5D), whereas culture of isolated CD8^+^ T cells with cisplatin in the absence of macrophages did increase CD8^+^ T cell IL-13 production (Figure 2F and Supplemental Figure 5E). In line with an earlier study (20), culture of BMDMs with IL-13 dose dependently increased IL-13 production (Supplemental Figure 5F).

**Figure 3:**
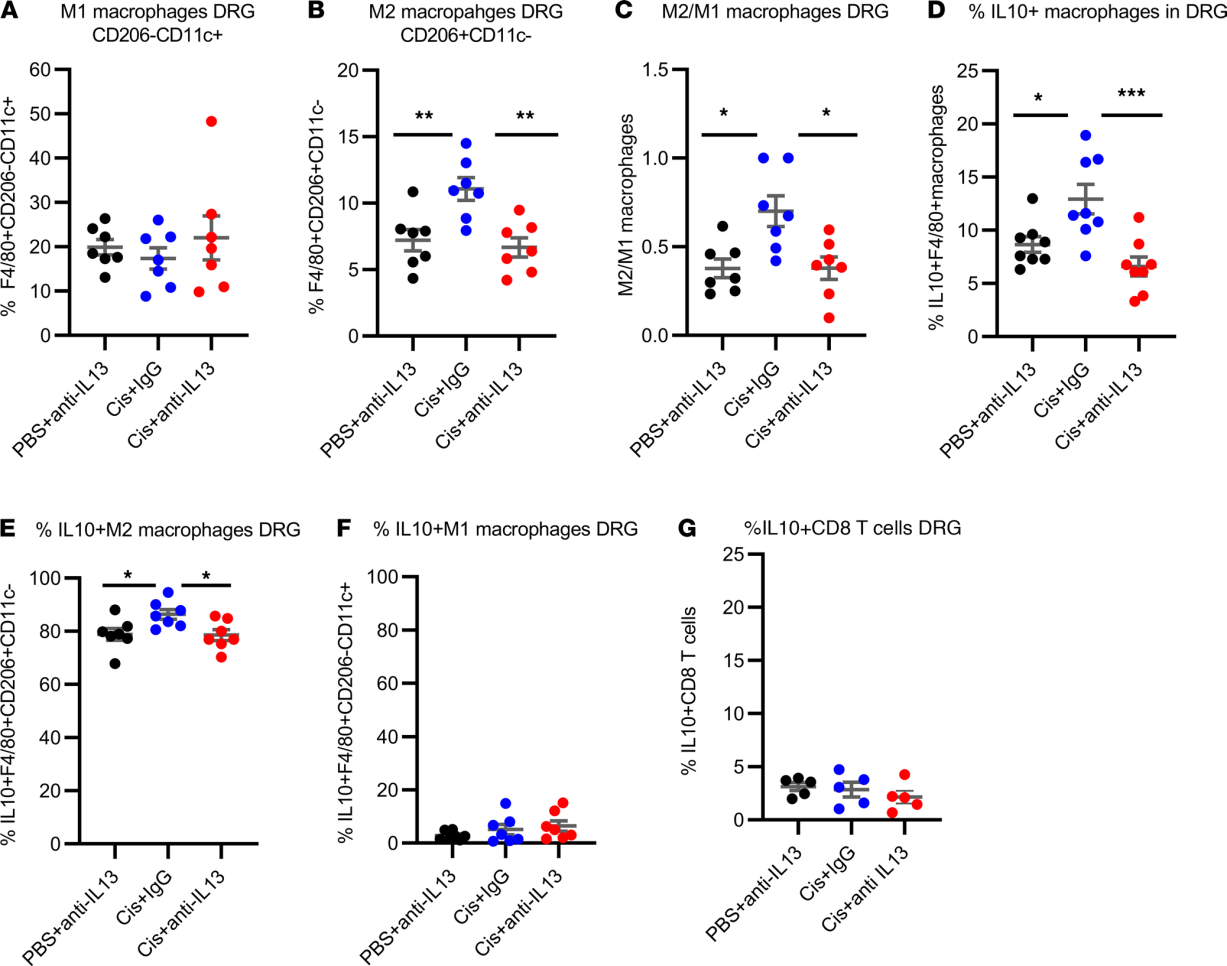
In vivo, IL-13 mediates M2 differentiation and IL-10 production by DRG macrophages in mice treated with cisplatin. Mice were treated with cisplatin (2 mg/kg i.p. on days 0, 1, and 2); on days 7 and 8 after the first dose of cisplatin, mice were injected intrathecally with anti–IL-13 antibody or control IgG (10 μg/mouse/d) and lumbar DRG isolated for flow cytometry. The percentage of (**A**) M1 (CD206^–^CD11c^+^), (**B**) M2 (CD206^+^CD11c^–^) macrophages within the F4/80^+^ population, and the (**C**) M2/M1 macrophage ratio are depicted. One-way ANOVA followed by Tukey’s multiple-comparison test. *P < 0.05, **P < 0.01; *n* = 4 male, 3 female/group. Percentage of (**D**) IL-10^+^F4/80^+^ macrophages, (**E**) IL-10^+^CD206^+^CD11c^–^ (M2) macrophages, (**F**) IL-10^+^CD206^–^CD11c^+^ (M1) macrophages in the DRG of mice treated with cisplatin followed by intrathecal administration of anti–IL-13 or control IgG. One-way ANOVA followed by Tukey’s multiple-comparison test. *P < 0.05, ***P < 0.001; *n* = 4 male, 3 female/group. (**G**) Percentage of IL-10^+^CD8^+^ T cells in DRG collected from mice treated with cisplatin or PBS followed by intrathecal anti–IL-13 or control IgG. One-way ANOVA: NS; *n* = 5 male/group. Data are shown as mean ± SEM.

Flow cytometric analysis of the DRG of WT mice showed that resolution of CIPN was associated with a shift toward M2 (CD206^+^CD11c^–^) macrophages without changes in M1 (CD206^–^CD11c^+^) macrophages (Figure 3, A–C, and Supplemental Figure 6A). Moreover, we detected an increase in macrophages producing IL-10 during resolution of CIPN (Figure 3D and Supplemental Figure 6B). Further analysis revealed that the increase in IL-10 in F4/80^+^ macrophages occurred in the M2 macrophage population (Figure 3E and Supplemental Figure 6C) and not in the M1 macrophage population (Figure 3F and Supplemental Figure 6D). Notably, blocking IL-13 signaling by intrathecal administration of anti–IL-13 prevented the increase in IL-10–producing DRG macrophages (Figure 3, D and E, and Supplemental Figure 6, B and C) and the shift in the M2/M1 ratio (Figure 3, A–C). Consistent with the findings in our in vitro study (Figure 2C), we did not detect a change in IL-10 production by DRG CD3^+^ T cells (Supplemental Figure 7, A and B) or the subset of CD8^+^ T cells in the DRG (Figure 3G). Collectively, these data indicated that cisplatin stimulated CD8^+^ T cells to produce IL-13, which promoted the production of IL-10 by DRG macrophages.

**Figure 4:**
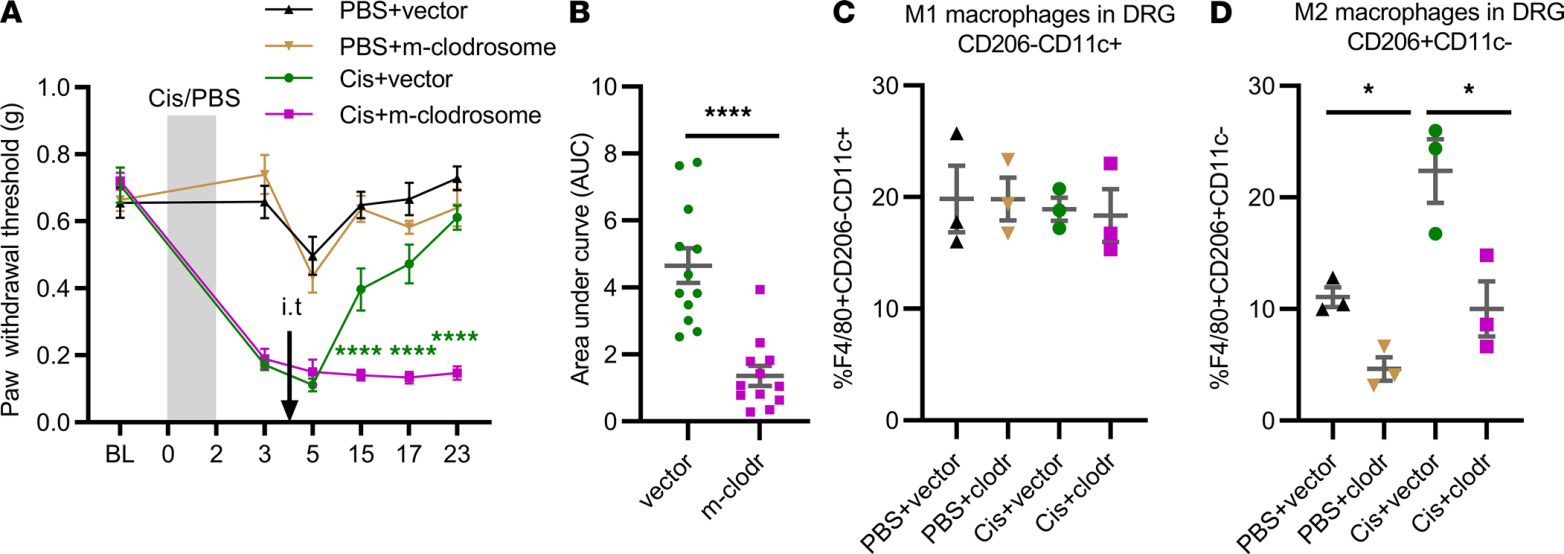
Intrathecal administration of mannosylated-clodrosome delays resolution of mechanical allodynia. (**A**) Male and female WT mice were treated with cisplatin (2 mg/kg i.p. on days 0, 1, and 2); on day 4 after the first dose of cisplatin they were injected intrathecally with clodronate-containing mannosylated liposomes or empty mannosylated liposomes (vector) as a control (10 μL/mouse) to deplete CD206^+^ macrophages. Mechanical allodynia was followed over time. Two-way repeated-measures ANOVA followed by Bonferroni’s multiple-comparison test. Cis+vector versus Cis+m-clodrosome; ****P < 0.0001; *n* = 8 male, 4 female/group. (**B**) AUC for the resolution phase (days 5–23) of the data in **A**. Welch’s *t* test; ****P < 0.001. (**C** and **D**) Effect of intrathecal administration of m-clodrosome on (**C**) M1(CD206^–^CD11c^+^) and (**D**) M2 (CD206^+^CD11c^–^) macrophages in the DRG as assessed by flow cytometry. Two-way repeated-measures ANOVA followed by Bonferroni’s multiple-comparison test; *P < 0.05; *n* = 3 female/group. All data are shown as mean ± SEM.

Next, we examined the effect of intrathecal injection of clodronate-containing mannosylated liposomes (m-clodrosome) to deplete local M2 macrophages (29) on the resolution of CIPN. The results (Figure 4, A and B) demonstrated that intrathecal administration of m-clodrosome delayed resolution of mechanical allodynia in cisplatin-treated male and female mice. Intrathecal administration of m-clodrosome reduced the percentage of M2 macrophages in the DRG without affecting M1 macrophages (Figure 4, C and D, and Supplemental Figure 8) and did not affect microglia in the spinal cord (29, 30). These findings identified a critical role of M2 macrophages in resolution of CIPN.

### TIM3 on CD8^+^ T cells is required for induction of IL-13 production by cisplatin.

**Figure 5:**
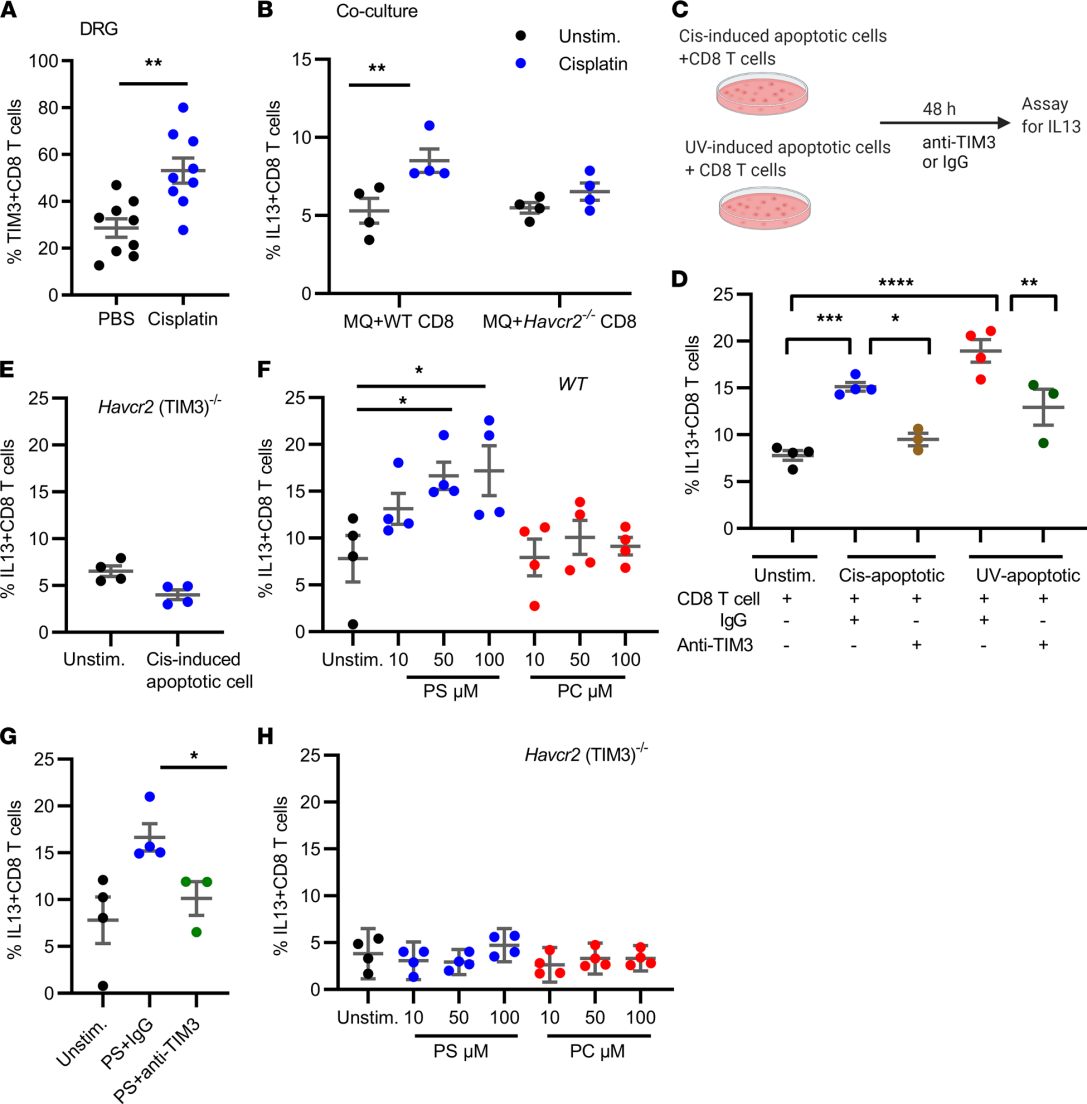
TIM3 on CD8^+^ T cells is required for induction of IL-13 production by cisplatin in vitro. (**A**) TIM3^+^CD8^+^ T cells in the DRG during resolution of cisplatin-induced CIPN. The percentage of TIM3^+^CD8^+^ T cells in the DRG was quantified by flow cytometry. Welch’s *t* test; **P < 0.001; *n* = 4 male, 5 female/group. (**B**) BMDMs were cocultured with either WT CD8^+^ T cells or *Havcr2* (TIM3)^–/–^ CD8^+^ T cells. Cocultures were treated with cisplatin (1 μg/mL) or left untreated (unstim.) and 48 hours later, CD8^+^ T cells were assayed for IL-13 by flow cytometry. Two-way ANOVA, Holm-Šidák multiple-comparison test, **P < 0.05; *n* = 4 independent experiments; 2 male, 2 female. (**C**) Schematic representation of coculture experiment assessing effect of apoptotic cells on IL-13 production by T cells. WT mouse splenocytes were treated with cisplatin (2.5 μg/mL) for 24 hours or were UV exposed for 10 minutes and cultured for 24 hours. Apoptotic cells were cocultured with WT CD8^+^ T cells (**D**) or *Havcr2* (TIM3)^–/–^ CD8^+^ T cells (**E**). Cells were assayed for IL-13^+^CD8^+^ T cells by flow cytometry 48 hours later. One-way ANOVA, Tukey’s multiple-comparison test, *P < 0.05, **P <0.01, ***P < 0.001, ****P < 0.0001; *n* = 3–4 independent experiments; 2 male, 1–2 female. (**F**) WT mouse splenocytes were treated with PS (phosphatidylserine) or PC (phosphatidylcholine as negative control) liposomes at increasing concentrations. *n* = 4 independent experiments, 2 male, 2 female, assayed for IL-13^+^CD8^+^ T cells. (**G**) Cultures of WT mouse splenocytes with PS liposomes (50 μM) were treated with anti-TIM3 neutralizing antibody or IgG as control. *n* = 3–4 independent experiments; 2 male, 1 female. One-way ANOVA, Dunnett’s multiple-comparison test, *P < 0.05. (**H**) *Havcr2* (TIM3)^–/–^ splenocytes were treated with PS or PC (as negative control) liposomes at increasing concentrations. *n* = 3 independent experiments; 3 female. After 48 hours, the percentage of IL-13^+^CD8^+^ T cells was assessed by flow cytometry. All data are shown as mean ± SEM.

TIM3 contains a PS binding pocket that allows for recognition of apoptotic cells exposing PS on their surface (24, 31), and cisplatin is known to induce apoptotic cell death of tumor cells and healthy dividing cells, including leukocytes (23, 27). We hypothesized that TIM3 on CD8^+^ T cells may contribute to the induction of IL-13 production by CD8^+^ T cells. In vivo, resolution of CIPN was associated with an increase in TIM3^+^CD8^+^ T cells in the DRG (Figure 5A and Supplemental Figure 9). To determine whether TIM3 on CD8^+^ T cells contributes to activation of the pain-resolving activity of CD8^+^ T cells, we used our in vitro coculture system. In these cultures, cisplatin increased IL-13 production by WT CD8^+^ T cells (Figure 5B and Supplemental Figure 10). However, we did not detect changes in IL-13 production by *Havcr2* (TIM3)^–/–^ CD8^+^ T cells (Figure 5B and Supplemental Figure 10). To determine whether apoptotic cells can activate IL-13 production by CD8^+^ T cells via a TIM3-dependent pathway, we used splenocytes treated with cisplatin or exposed to UV radiation as a source of apoptotic cells with PS exposed on the cell surface (Figure 5C and Supplemental Figure 11). Coculture of CD8^+^ T cells with cisplatin-induced or UV-induced apoptotic splenocytes upregulated IL-13 production by CD8^+^ T cells (Figure 5D and Supplemental Figure 11C). Addition of a neutralizing antibody to block TIM3 to these cultures prevented the increase in IL-13–producing CD8^+^ T cells (Figure 5D and Supplemental Figure 11C). In addition, we did not detect induction of IL-13 in *Havcr2* (TIM3)^–/–^ CD8^+^ T cells (Figure 5E and Supplemental Figure 11D).

To investigate whether exposure of TIM3^+^CD8^+^ T cells to PS is sufficient to induce IL-13, we used PS liposomes. The results (shown in Figure 5F and Supplemental Figure 12, A–C) demonstrated that PS liposomes dose dependently increased the production of IL-13 by CD8^+^ T cells, whereas there was no effect of control phosphatidylcholine liposomes. Notably, addition of anti-TIM3 neutralizing antibody prevented the PS-induced increase in IL-13 production by CD8^+^ T cells (Figure 5G and Supplemental Figure 12C). Moreover, PS liposomes did not induce IL-13 production by *Havcr2* (TIM3)^–/–^ CD8^+^ T cells (Figure 5H and Supplemental Figure 12D). These findings indicated that PS expressed on the surface of apoptotic cells stimulated TIM3 on CD8 cells, leading to increased production of IL-13.

### TIM3 on CD8^+^ T cells is required for resolution of mechanical allodynia induced by cisplatin.

**Figure 6:**
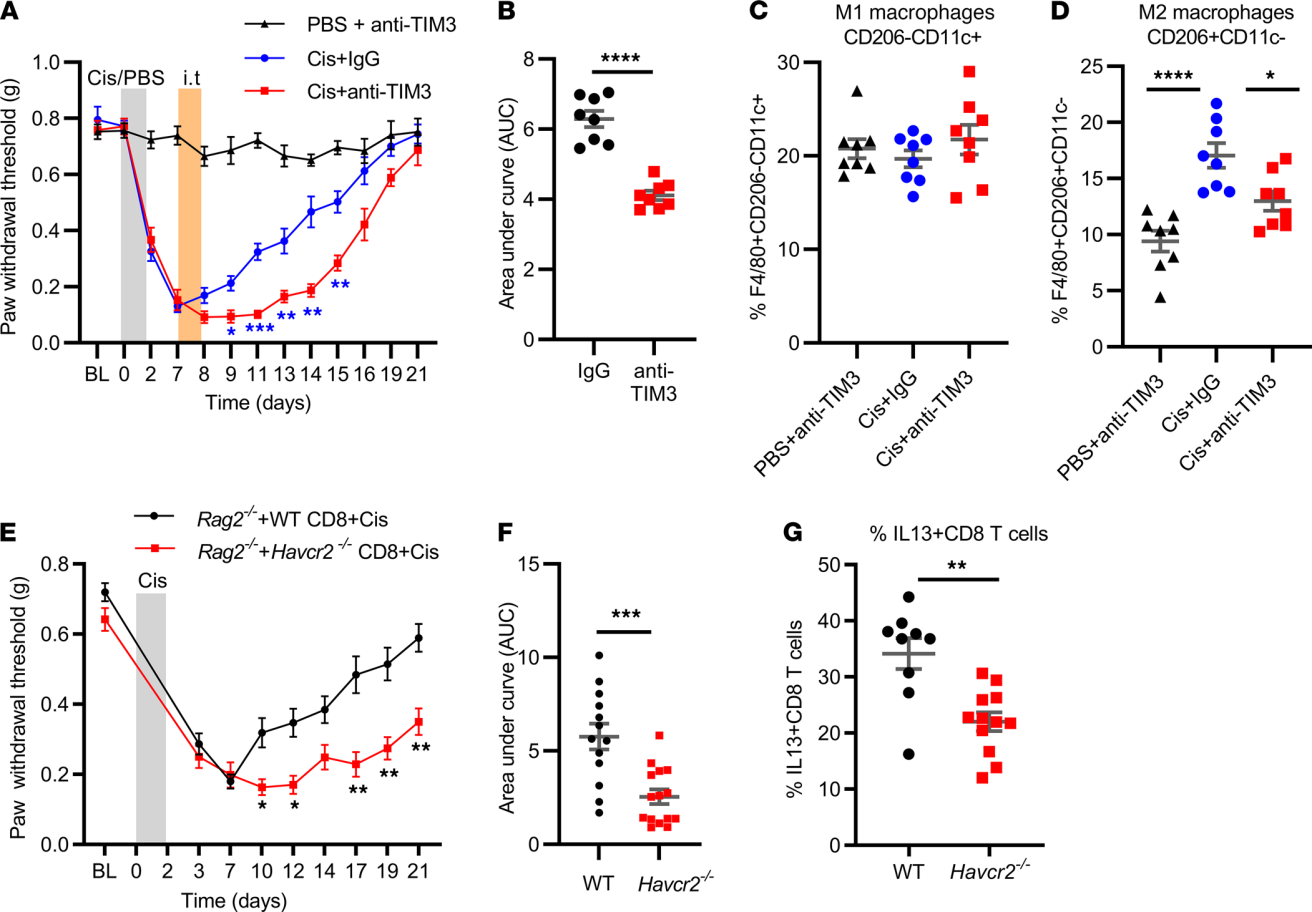
In vivo, TIM3^+^CD8^+^ T cells promote resolution of CIPN. (**A**) Male and female mice were treated with cisplatin (2 mg/kg i.p. on days 0, 1, and 2); on days 7 and 8 after the first dose of cisplatin, they were intrathecally injected with anti-TIM3 antibody or control IgG (10 μg/mouse/d) and mechanical allodynia was followed over time. Two-way repeated-measures ANOVA followed by Bonferroni’s multiple-comparison test for Cis+IgG versus Cis+anti-TIM3. *P < 0.05, **P < 0.01, ***P < 0.001; *n* = 4 male, 4 female/group. (**B**) AUC of the resolution phase (days 8–21) of the cisplatin-treated mice in **A**. Welch’s *t* test; ****P < 0.000.1 (**C**) Percentage of M1 (CD206^–^CD11c^+^) and (**D**) M2 (CD206^+^CD11c^–^) macrophages in the DRG of mice treated with cisplatin followed by anti-TIM3 or IgG as in **A**. One-way ANOVA followed by Dunnett’s multiple-comparison test; ****P < 0.0001, *P < 0.05; *n* = 4 male, 4 female/group. (**E**) Time course of change in mechanical sensitivity in male and female *Rag2*^–/–^ mice reconstituted with either *Havcr2* (TIM3)^–/–^ CD8^+^ T cells or WT CD8^+^ T cells. Ten days after reconstitution, mice were treated with cisplatin (2 mg/kg i.p. on days 0, 1, and 2), and mechanical allodynia was monitored over time. Two-way repeated-measures ANOVA followed by Bonferroni’s multiple-comparison test. *P < 0.05, **P < 0.01; *Rag2*^–/–^+WT CD8+Cis *n* = 8 male, 5 female; *Rag2*^–/–^+*Havcr2* (TIM3)^–/–^ CD8+Cis *n* = 8 male, 7 female. (**F**) AUC of the resolution phase (days 7–21) of the data in **C**; Welch’s *t* test; ***P < 0.01. (**G**) Percentage of IL-13^+^CD8^+^ T cells in DRG of *Rag2*^–/–^ mice reconstituted with either *Havcr2* (TIM3)^–/–^ CD8^+^ T cells or WT CD8^+^ T cells treated with cisplatin. Welch’s *t* test; **P < 0.01. *n* = 4–7 male, 5 female/group. All data are shown as mean ± SEM.

To identify the in vivo contribution of TIM3 to resolution of CIPN, we treated male and female WT mice with cisplatin followed by intrathecal administration of a neutralizing anti-TIM3 antibody on days 7 and 8 after the first dose of cisplatin. Anti-TIM3 treatment significantly delayed resolution of mechanical allodynia compared with mice treated with control IgG in both sexes (Figure 6, A and B). Delayed resolution of CIPN in mice receiving intrathecal administration of anti-TIM3 was associated with a reduction in M2 macrophages in the DRG as compared with mice treated with control IgG (Figure 6D and Supplemental Figure 13, A and B). DRG M1 macrophages were not affected by intrathecal administration of anti-TIM3 (Figure 6C and Supplemental Figure 13, A and B).

To determine whether CD8^+^ T cells need to express TIM3 in order to promote resolution of allodynia, we compared resolution of cisplatin-induced allodynia in male and female *Rag2*^–/–^ mice reconstituted with WT or *Havcr2* (TIM3)^–/–^ CD8^+^ T cells (Figure 6, E and F). The results showed that resolution of cisplatin-induced allodynia was significantly delayed in *Rag2*^–/–^ mice reconstituted with TIM3-deficient CD8^+^ T cells as compared with WT CD8^+^ T cells (Figure 6, E and F). The finding that resolution was delayed but not completely absent in *Rag2*^–/–^ mice reconstituted with *Havcr2* (TIM3)^–/–^ CD8^+^ T cells indicated that TIM3 signaling does not represent the only pathway via which CD8^+^ T cells can be activated to promote resolution of CIPN.

The delayed resolution of CIPN in *Rag2*^–/–^ mice reconstituted with TIM3-deficient CD8^+^ T cells was associated with a significant reduction in the percentage of IL-13–producing CD8^+^ T cells in the DRG of these mice (Figure 6G). Supplemental Figure 14 shows that there were no differences in CD8^+^ T cell reconstitution of the spleen and DRG between *Rag2*^–/–^ mice receiving WT or TIM3-deficient CD8^+^ T cells.

## Discussion

The endogenous cellular mechanisms underlying the resolution of pain have only begun to be investigated. We proposed recently that understanding the contribution of the immune system to pain resolution may identify potentially novel targets for treatment (2, 4). Here, we used a transient model of chemotherapy-induced peripheral neuropathic pain to unravel the pathways involved in the critical contribution of the immune system to resolution of mechanical allodynia. Our previous studies have shown that CD8^+^ T cells and IL-10 are both required for CIPN resolution but that the CD8^+^ T cells are not the source of the IL-10. Our current findings provide the missing link by demonstrating that CD8^+^ T cells produce IL-13 in response to activation of TIM3 on CD8^+^ T cells by PS expressed on the surface of cells damaged by chemotherapy. In turn, IL-13 increases IL-10 production by local macrophages, leading to resolution of CIPN.

IL-13 is mainly known for its contribution to allergy and asthma and shares functional properties with IL-4 because it signals via receptor complexes composed of the α subunit of the IL-4 receptor (IL-4 Rα) and the IL-13 binding subunit IL-13 Rα1 (32). Not much is known about a potential role of IL-13 in pain. Clinically, plasma IL-13 levels are inversely correlated with the severity of pain in amputees with residual limb pain (33, 34), indicating a potential role of IL-13 in suppression of pain. One previous study found that perineurial administration of IL-13 decreased mechanical allodynia after partial sciatic nerve ligation, and this was attributed to a decrease in macrophage proinflammatory cytokine production (35). Here, we uncovered a thus far unappreciated role of CD8^+^ T cell–derived IL-13 in pain resolution by showing that (a) intrathecal administration of anti–IL-13 delayed resolution of pain in mice treated with cisplatin and (b) *Il13*^–/–^ CD8^+^ T cells did not promote resolution of pain when transferred to T cell–deficient *Rag2*^–/–^ mice, whereas WT CD8^+^ T cells did. In searching for the pathway via which CD8^+^ T cells are activated to produce IL-13 during resolution of cisplatin-induced mechanical allodynia, we focused on the checkpoint molecule TIM3. Expression of TIM3 on T cells has been associated with acquisition of a dysfunctional exhausted phenotype, especially during viral infections (36, 37). However, accumulating evidence indicates that TIM3 also contributes to effector T cell responses (36, 37). We identified a potentially novel role of TIM3 on CD8^+^ T cells by showing that TIM3-deficient CD8^+^ T cells failed to promote resolution of CIPN. Moreover, resolution of CIPN was associated with an increase in TIM3^+^CD8^+^ T cells in the DRG.

Cisplatin induces damage and apoptotic cell death of cancer cells and healthy tissue (38, 39). TIM3 has a binding pocket that recognizes PS, a cell membrane phospholipid that translocates from the inner to the outer leaflet of the cell membrane during apoptosis (24, 31). The interaction of PS with the TIM3 on phagocytic cells is important for removal of apoptotic cells (31). However, the functional consequences of the interaction between PS and TIM3 on CD8^+^ T cells has not yet been unraveled. Our finding that apoptotic cells or PS liposomes induced IL-13 production by CD8^+^ T cells via TIM3 fills this knowledge gap. Moreover, in vivo, we showed that TIM3–expressing CD8^+^ T cells are required for the increase in IL-13 production by these cells and for the resolution of CIPN. Thus, our study identified a role of TIM3 in the induction of IL-13 production by CD8^+^ T cells in response to cell damage and ultimately resolution of pain.

On the basis of our current and previous studies, we propose that the IL-13–dependent resolution of pain in cisplatin-treated mice is mediated via IL-10 produced by macrophages. The prevailing idea is that macrophages promote tissue damage, and there is ample evidence that activated macrophages promote and maintain chronic pain (40). However, there is increasing evidence for a dual role of macrophages depending on their phenotype and activation stage; M1 macrophages can increase damage and pain (40), while M2-type macrophages promote tissue healing and pain resolution (2, 4, 20, 29). We showed previously that resolution of CIPN is delayed in mice deficient in IL-10 or after intrathecal administration of anti–IL-10 antibody in WT mice (15, 17). Moreover, adoptive transfer studies showed that *Il10*^–/–^ CD8^+^ T cells promote resolution of CIPN as effectively as WT CD8^+^ T cells, indicating that CD8^+^ T cells are not the source of the IL-10 needed for resolution (15, 17). Here we showed that in vitro, cisplatin increased IL-10 production by macrophages via a pathway dependent on IL-13 production by CD8^+^ T cells. Moreover, in vivo, resolution of CIPN was associated with an increase in IL-10–producing macrophages, and this increase was prevented by inhibition of IL-13 signaling. These findings are in line with a recent study showing that IL-13 (in this case derived from CD4^+^ Tregs) increased IL-10 production by macrophages and resolution of inflammation in a model of peritonitis and in a model of lung inflammation (20). Therefore, our current findings may have broader implications for understanding the interaction between T cells and macrophages in the context of resolution of pain and inflammation as well as tissue healing.

The pain-reducing effects of exogenously administered IL-10 and of IL-10 produced by macrophages have been attributed to suppression of proinflammatory cytokine release by spinal cord microglia (29, 41). IL-10 is known to reprogram macrophage metabolism, thereby amplifying antiinflammatory and resolution functions (42). However, microglia and macrophages are not the only potential targets via which IL-10 can promote resolution. Recent studies by us and others have shown that IL-10 can also directly signal to sensory neurons via IL-10 receptors in these cells (15, 16, 43). Notably, genetic deletion of IL-10 receptors from sensory neurons delays resolution of neuropathic pain, identifying a role of these neuronal IL-10 receptors in pain resolution (17, 44). Ex vivo, IL-10 suppresses the spontaneous activity of DRG neurons isolated from rodents with CIPN (15, 16) and reduces excitability of DRG neurons from control mice (44). The reduction in DRG neuron hyperexcitability and spontaneous activity in response to IL-10 can be mediated via changes in NMDA receptor phosphorylation (44) and in expression of ion channels (45). IL-10 is also known to increase mitophagy of damaged mitochondria (43). We have shown that cisplatin decreases mitochondrial health in DRG neurons and that prevention of this mitochondrial damage prevents cisplatin-induced peripheral neuropathy (46, 47). It remains to be determined whether endogenous IL-10 signaling contributes to normalization of DRG neuron mitochondrial health in cisplatin-treated animals recovering from neuropathic pain.

Based on our current and previous findings, we propose that TIM3 expressed on CD8^+^ T cells binds to PS exposed because of cellular damage by cisplatin, resulting in IL-13 production by CD8^+^ T cells. IL-13 stimulates the production of IL-10 by macrophages leading to a reduction in the excitability and spontaneous activity of peripheral sensory neurons and resolution of CIPN. Interventions that activate this CD8^+^ T cell/TIM3/IL-13-to-macrophage/IL-10 pathway to boost endogenous pain resolution could provide a treatment for persistent CIPN, thereby improving quality of life of the growing number of cancer survivors.

## Methods

### Animals.

Adult (8–12 weeks of age) male and female C57BL/6J mice and *Rag2*^–/–^, in a C57BL/6J background, were obtained from The Jackson Laboratory (stock number 008449). *Il13*^–/–^ and *Havcr2* (TIM3)^–/–^ mice were provided by Andrew McKenzie, MRC, Cambridge, United Kingdom. Mice were housed in a 12-hour light/12-hour dark cycle at room temperature and fed ad libitum. Animals were randomly assigned to the groups and investigators were blinded to treatment. All animal experiments were performed at the University of Texas Health MD Anderson Cancer Center, Houston, Texas, according to procedures approved by the IACUC.

### Induction of CIPN.

To induce CIPN, mice were i.p. treated with cisplatin (Teva Pharmaceutical Industries Ltd.) for 3 days at a dose of 2 mg/kg/d, for a cumulative dose of 6 mg/kg (16). PBS was used as vehicle.

### Intrathecal injections.

Anti–IL-13 (R&D Systems, catalog AF-413-NA), anti-TIM3 (BioLegend, catalog 119712), or normal IgG (R&D Systems, catalog AB-108-C) was administered intrathecally at a dose of 10 μg/mouse/d in a volume of 5 μL on days 7 and 8 after the first dose of cisplatin under isoflurane anesthesia as described previously (48). Earlier studies have shown that proteins injected intrathecally enter both the DRG and spinal cord (49). For macrophage depletion, mice were intrathecally injected on day 4 after the first dose of cisplatin under isoflurane anesthesia with 10 μL of either empty liposomes or clodronate-containing liposomes from the mannosylated macrophage depletion kit (catalog CLD-8914, Encapsula Nano Sciences) (29).

### Adoptive transfer.

Adoptive transfer of CD8^+^ T cells was performed before chemotherapy injection, as previously described (15, 16). Spleens were collected from WT, *Il13*^–/–^, and *Havcr2*^–/–^ mice, and single-cell suspensions were obtained by passing the spleens through a 70 μm mesh. CD8^+^ T cells were isolated using a negative selection kit (Miltenyi Biotec, catalog 130-104-075), according to the manufacturer’s instructions. Three million CD8^+^ T cells in 100 μL PBS plus 0.1% BSA or vehicle were i.v. injected into the tail vein. Reconstitution was confirmed by flow cytometric analysis of CD8^+^ T cells in the spleen and DRG.

### Von Frey test for mechanical allodynia.

Mechanical allodynia as a readout for CIPN was measured as the hind paw withdrawal response to von Frey hair stimulation by an investigator blinded to group and treatment using the up-and-down method, as we described previously (15, 50). Mice were placed in a Plexiglas enclosure (10 × 10 × 13 cm^3^) with a mesh floor for 30 minutes before testing. A series of von Frey hairs (0.02, 0.07, 0.16, 0.4, 0.6, 1.0, and 1.4 g; Stoelting) were applied perpendicular to the midplantar surface of the hind paw. A trial began with the application of the 0.16 g hair using the up-and-down method as described previously (51).

### Flow cytometry.

For flow cytometric analysis of leukocytes, lumbar DRG (L4-6) were collected at the end of the experiment (days 21–23). Tissue was incubated in digestion buffer (RPMI with 10 mM HEPES, 5 mg/mL BSA, 1.6 mg/mL collagenase) at 37°C with gentle agitation and triturated thrice (2 times with a 1 mL pipette tips; then once with 22G needle) each after 20 minutes of incubation. The cell suspension was passed through a 70 μm cell strainer, then spun down at 300*g* for 5 minutes at room temperature, and cells were resuspended in 1 mL of RPMI media (Corning, catalog 10-041-CV) supplemented with 10% heat-inactivated FBS (MilliporeSigma, catalog F4135) and 1× of penicillin streptomycin l-glutamine (Corning, catalog 30-009). For intracellular cytokine staining, cells were stimulated with Brefeldin A 1 μL/mL (BioLegend, 420601) for 4 hours. Cells were washed with wash buffer (PBS with 2% heat-inactivated FBS) and incubated with live/dead fixable dead cell dye (Invitrogen, catalog L34969) followed by surface staining using antibodies directed against CD45 Brilliant Violet 570 (BioLegend, clone 30-F11), F4/80 PB (BioLegend, clone BM8), TIM3 BV421 (BioLegend, clone RMT3-23), and CD8 AF700 (BioLegend, clone 53-6.7) for 30 minutes at 4°C. After surface staining, cells were fixed and permeabilized using the mouse Foxp3 buffer set (BD Pharmingen, catalog 560409) according to the manufacturer’s protocol. The cells were subsequently stained intracellularly with IL-10 Alexa Fluor 647 (BioLegend, clone JES5-16E3) and IL-13 PE (eBioscience, clone 85BRD). Cells were resuspended in wash buffer and acquired on Gallios flow cytometer (Beckman Coulter) at the University of Texas MD Anderson Cancer Center Flow Cytometry & Cellular Imaging Core Facility.

To check the reconstitution of CD8^+^ T cells in the spleen by flow cytometric analysis, the spleens were disaggregated by gentle pressure using a 1 mL syringe and then passed through 70 μm cell strainer filter. Cells were washed with wash buffer and pelleted. RBCs were lysed by addition of 2 mL RBC lysis buffer (BioLegend, catalog 420301), mixed gently to resuspend cells, and incubated for 4 minutes on ice, followed by wash buffer wash and spin. Cells were surface-stained using antibodies directed against CD45 Brilliant Violet 570 (BioLegend, clone 30-F11), CD3 PE Cy5 (BioLegend, clone 17A2), and CD8 AF700 (BioLegend, clone 53-6.7) for 30 minutes at 4°C followed by wash buffer wash and spin. Cells were resuspended in wash buffer and acquired on Gallios flow cytometer. Data for all flow cytometric evaluations were analyzed using Kaluza 2.1.1 software (Beckman Coulter).

### Differentiation of BMDMs and coculture with CD8^+^ T cells.

Bone marrow cells from 8- to 12-week-old male or female mice were cultured in DMEM (Corning, catalog 10-013) supplemented with 10% heat-inactivated FBS, 1× of penicillin streptomycin l-glutamine, and 10 μg/mL of macrophage colony-stimulating factor (Miltenyi Biotec, catalog 130-101-706). Adherent monocytes were left to differentiate into macrophages for 5 days with medium change once after 3 days of culture (52). BMDMs were harvested and plated in 48-well plates (2 × 10^5^ cells per well) a day before coculturing with CD8^+^ T cells isolated from the spleen in a 1:1 ratio. Anti–IL-13 (1 μg/mL; R&D Systems, catalog AF-413-NA) or control IgG (R&D Systems, catalog AB-108-C) were added in some wells. Forty-eight hours later, cells were collected for flow cytometric analysis of intracellular IL-10.

### Liposome preparation and treatment.

Liposomes were prepared as described previously (53) with slight modifications. Briefly, PS (Avanti Polar Lipids, 383907-32-2) was dissolved in ethanol/water (1:1); phosphatidylcholine (Avanti Polar Lipids, 97281-44-2) and cholesterol (MilliporeSigma, C3045) were dissolved in ethanol using water bath sonicator at 40°C. PS or phosphatidylcholine and cholesterol were mixed in 80:20 molar ratio followed by complete evaporation at 40°C using a magnetic stir plate in a biosafety cabinet. The dehydrated lipid/cholesterol mix was resuspended in PBS to a final concentration of 2 mM and then allowed to swell for 4 hours at room temperature. Liposomes were then sonicated using a water bath sonicator for clarity and desired size (150–200 nm). The liposomal size was confirmed using Zetasizer Nano (Malvern Panalytical).

Mouse splenocytes (1 × 10^6^/well) were plated in a 24-well tissue culture plate and treated with an increasing concentration of PS or phosphatidylcholine (10 μM, 50 μM, 100 μM). Anti–Tim-3 (1 μg/mL; BioLegend, catalog 134002) or control IgG was added in some wells, and 48 hours later, cells were harvested for flow cytometric analysis of intracellular IL-13.

### Preparation of apoptotic cells and coculture with CD8^+^ T cells.

For preparing apoptotic cells, splenocytes were incubated with 2.5 μg/mL of cisplatin in DMEM supplemented with 10 % heat-inactivated FBS and 1× of penicillin streptomycin l-glutamine for 24 hours or exposed to UV light for 10 minutes (240 J/s/m^2^) and incubated for 24 hours in the same medium. Apoptosis was confirmed by annexin V-FITC and propidium iodide staining (BD Pharmingen, 556547) followed by flow cytometric analysis. After washing, cisplatin-induced and UV-induced apoptotic cells (0.5 × 10^6^ cells/well) were cocultured with CD8^+^ T cells in a 1:1 ratio in 24-well plates. Anti-TIM3 or control IgG was added in some wells, and 48 hours later, intracellular IL-13 was analyzed by flow cytometric analysis.

### Statistics.

Data were analyzed using 2-tailed Welch’s *t* test for 2 groups with 1 variable tested, 1-way ANOVA followed by Tukey’s multiple-comparison test for multiple groups with only 1 variable tested, 2-way ANOVA with Holm-Šidák posttests for more than 2 groups with multiple variables tested, or repeated-measures 2-way ANOVA with Bonferroni’s correction for multiple tests. To facilitate statistical analysis of group differences in resolution of mechanical allodynia, we calculated the AUC of the recovery phase using the following equation: ΔX*([Y1 + Y2]/2) – baseline, where ΔX is the curve between 2 X values, Y1 is Y value at the lowest X, and Y2 is Y value at the highest X, in GraphPad Prism 9. Data are shown as mean ± SEM. A *P* value less than 0.05 was considered statistically significant.

### Study approval.

All procedures were consistent with the NIH’s *Guide for the Care and Use of Laboratory Animals* (National Academies Press, 2011) and the Ethical Issues of the International Association for the Study of Pain (54) and were approved by the IACUCs of University of Texas M.D. Anderson Cancer Center.

## Author contributions

SKS, AK, and CJH conceptualized and designed the research. SKS, KK, GOL, DW, and JFA performed the experiments. SKS, AK, and CJH interpreted the results of the experiments and wrote the paper; all authors edited the manuscript.

## Supplementary Material

Supplemental data

## Version 1 03/08/2022

Electronic publication
