# Supplementary material for: CD8+ T cell–derived IL-13 increases macrophage IL-10 to resolve neuropathic pain
Source: JCI Insight. 2022 Mar 8;7(5):e154194. doi: 10.1172/jci.insight.154194 (PMC8983134; doi:10.1172/jci.insight.154194)
Supplement: Supplemental data [file jciinsight-7-154194-s122.pdf]

Supplemental Figures

CD8 T cell-derived IL13 increases macrophage IL10 to resolve neuropathic pain

Susmita K. Singh[1], Karen Krukowski[1,2], Geoffroy O. Laumet[1,3], Drew Weis[1],Jenolyn Alexander[1], Cobi J. Heijnen[1], Annemieke Kavelaars[1]

Supplemental Figure 1

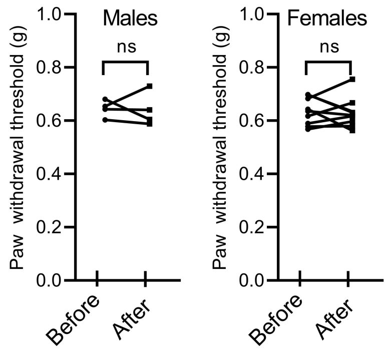

Supplemental Figure 1. Anti-IL13 does not affect pain sensitivity in PBS treated mice. Mean paw withdrawal threshold before (average of day 2,4,6) and after (Average of day 8,10, 12, 15) intrathecal administration of anti-IL13 to PBS treated male and female mice depicted in figure 1A. Data were analyzed by paired T-test.

Supplemental Figure 2

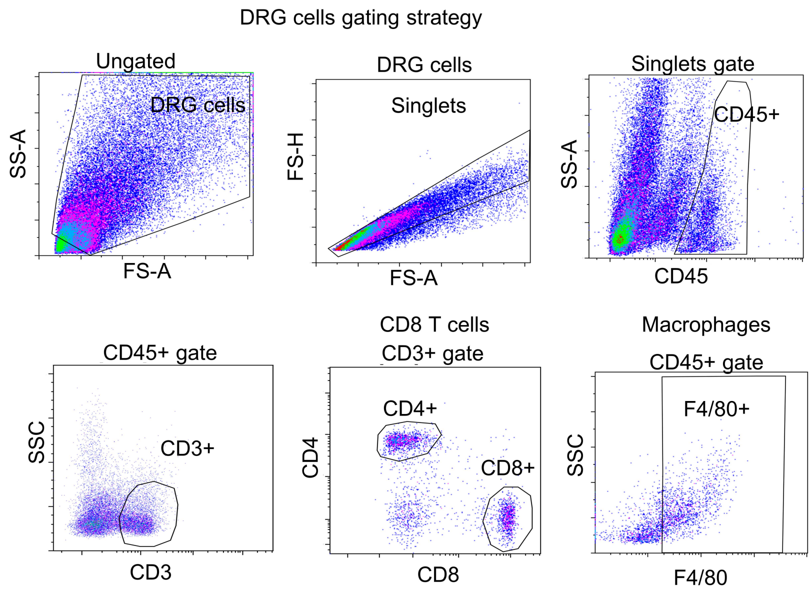

Supplemental Figure 2. Gating strategy for analysis of T cells and macrophages in the DRG of cisplatin-treated mice. Singlets were selected, followed by gating for CD45+ leukocytes and selection of CD8+ T cells and F4/80+ macrophages

Supplemental Figure 3

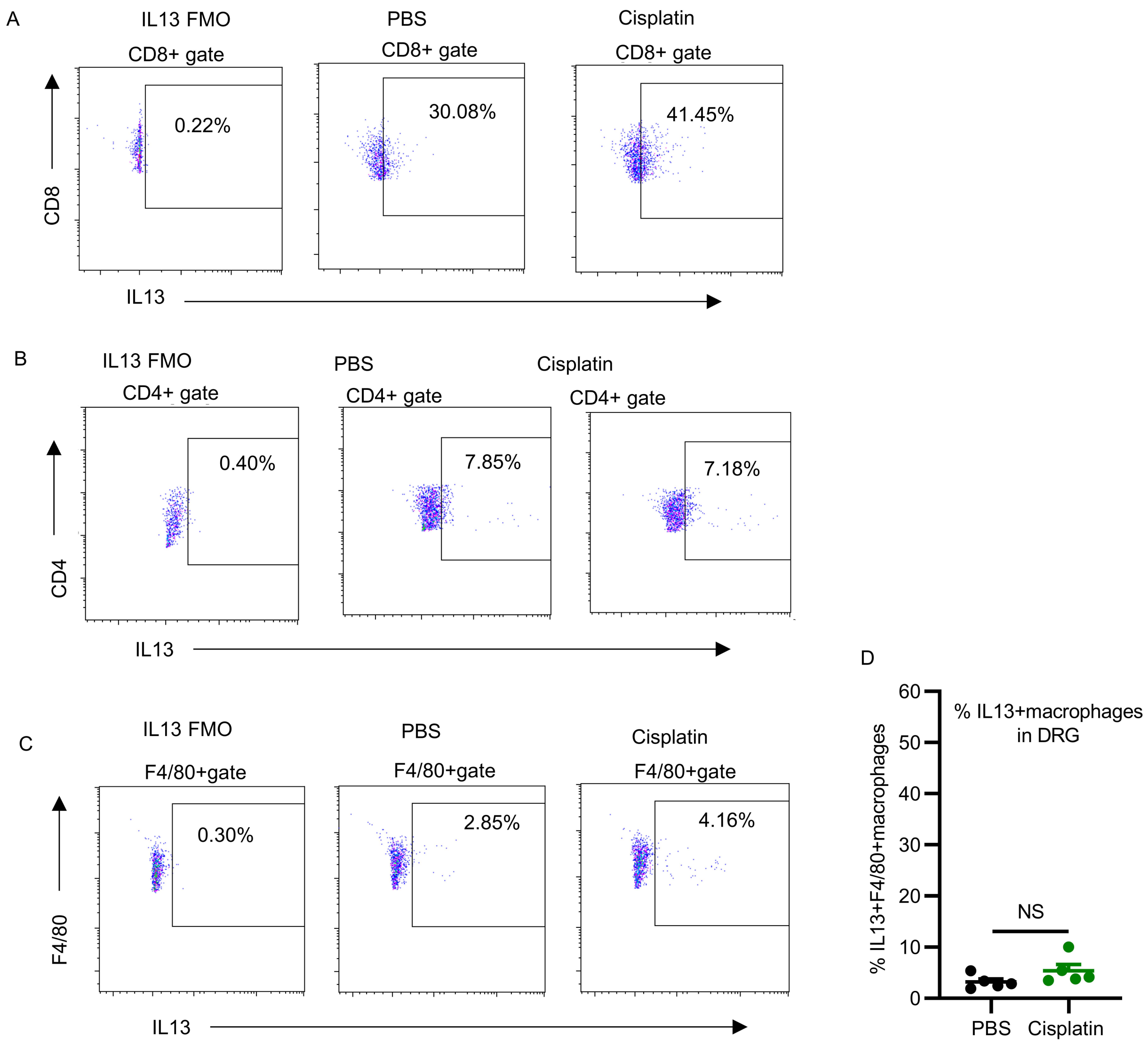

Supplemental Figure 3. IL13-positive cells in the DRG of cisplatin-treated mice. **(A)** FMO for this experiment as assessed in splenocytes and representative examples of the identification of IL13+CD8 T cells corresponding to the data presented in figure 1D. **(B)** FMO and representative example of the identification of IL13+CD4 T cells in the DRG corresponding to the data presented in Figure 1E. **(C)** FMO and representative example of the identification of IL13+ macrophages in the DRG and **(D)** Quantification of these data in n=5M/group.

Supplemental Figure 4

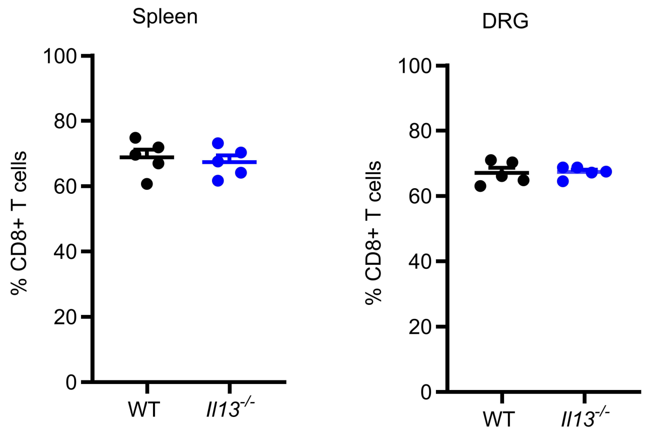

Supplemental Figure 4. Reconstitution of *Rag2*$^{-/-}$ mice with CD8 T cells. Analysis of CD8 T cells in the spleen and DRG of *Rag2*$^{-/-}$ mice reconstituted with WT or *Il13*$^{-/-}$ CD8 T cells. Data represent % CD8+T cells within the CD45+ population. n= 3M+2F/group; Welch's T-test: ns.

Supplemental Figure 5

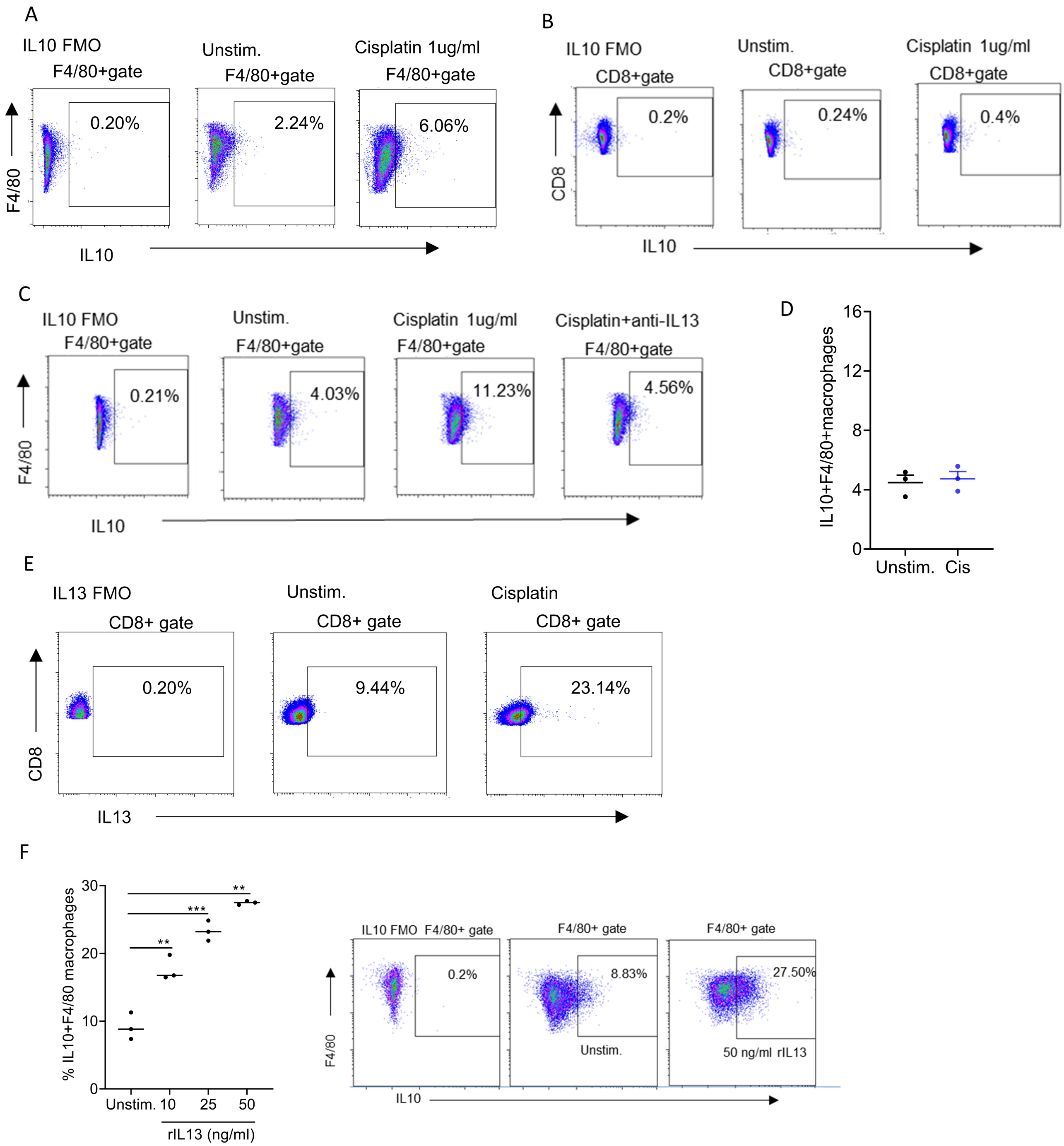

Supplemental Figure 5. IL10 production in cultures of BMDM and CD8T cells. (A-C) Representative flow cytometry plot for the data presented in Figure 2A, B and C. **(D)** Cisplatin does not induce macrophage IL10 production in the absence of CD8 T cells. Cultures of bone marrow-derived macrophages (BMDMs) were treated with cisplatin (1µg/ml) or left untreated (unstim.) and 24 h later the cells were assayed for IL10 containing F4/80+ macrophages by flow cytometry. n=3 males. **(E)** Representative flow cytometry plot for data in Figure 2F. **(F)** IL13 dose-dependently increases macrophage IL10 production. BMDM were treated with rIL13 overnight and assayed for IL10 by flow cytometry. n=3F. One-way ANOVA followed by Dunnett's multiple comparisons test, **p<0.01, ***p<0.001.

Supplemental Figure 6

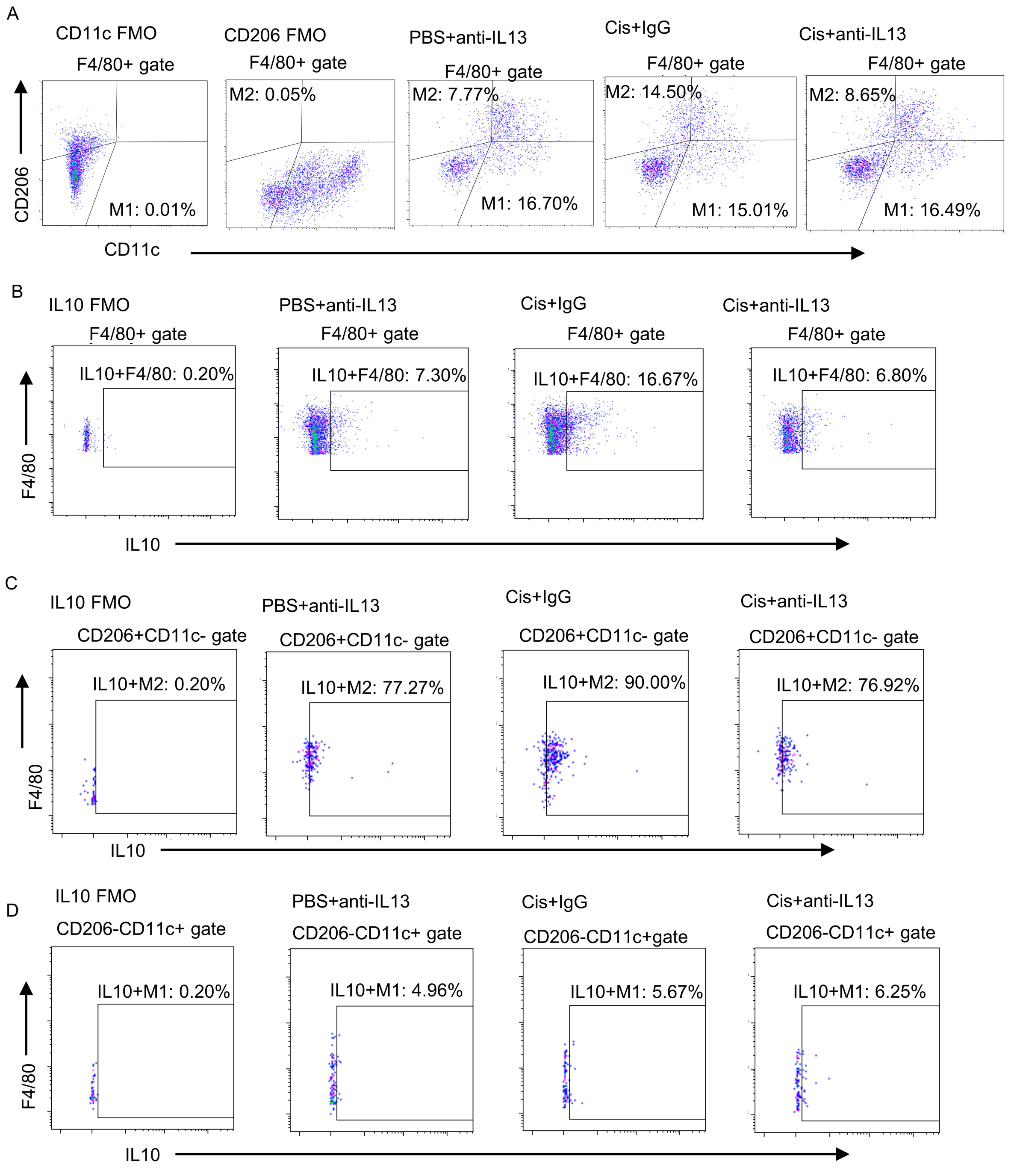

Supplemental Figure 6. Flowcytometry plots of M1, M2 and IL10 producing cells in the DRG of cisplatin-treated mice. Macrophages and CD8 T cells were gated as in Supplementary Figure 2. **(A)** FMO for CD206 and CD11c as assessed in splenocytes and representative examples for the identification of M2 (CD206+CD11c-) and M1 (CD206-CD11c+) macrophages (corresponding to Figure 3A, B). **(B)** FMO for IL10+ macrophages and representative examples of identification of IL10+ macrophages (corresponding to figure 3D). **(C)** FMO for IL10+ macrophages and representative examples of identification of IL10+ M2 macrophages (corresponding to figure 3E). **(D)** FMO for IL10+ macrophages and representative examples of identification of IL10+ M1 macrophages (corresponding to figure 3E).

Supplemental Figure 7

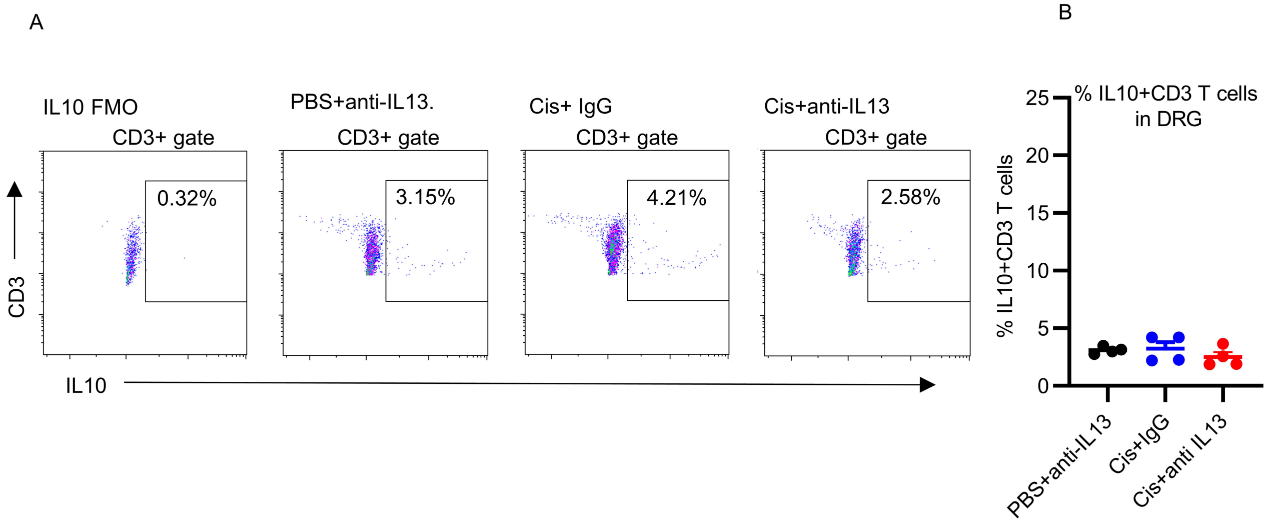

Supplemental Figure 7. Flowcytometry plots of IL10+CD3 T cells in the DRG of cisplatin-treated mice. (A) CD3 T cells were gated as in Supplementary Figure 2. FMO for IL10+CD3 T cells and representative examples of identification of IL10+CD3 T cells. **(B)** Quantification of IL10+CD3 T cells in DRG. n=4M/group.

F

Supplemental Figure 8

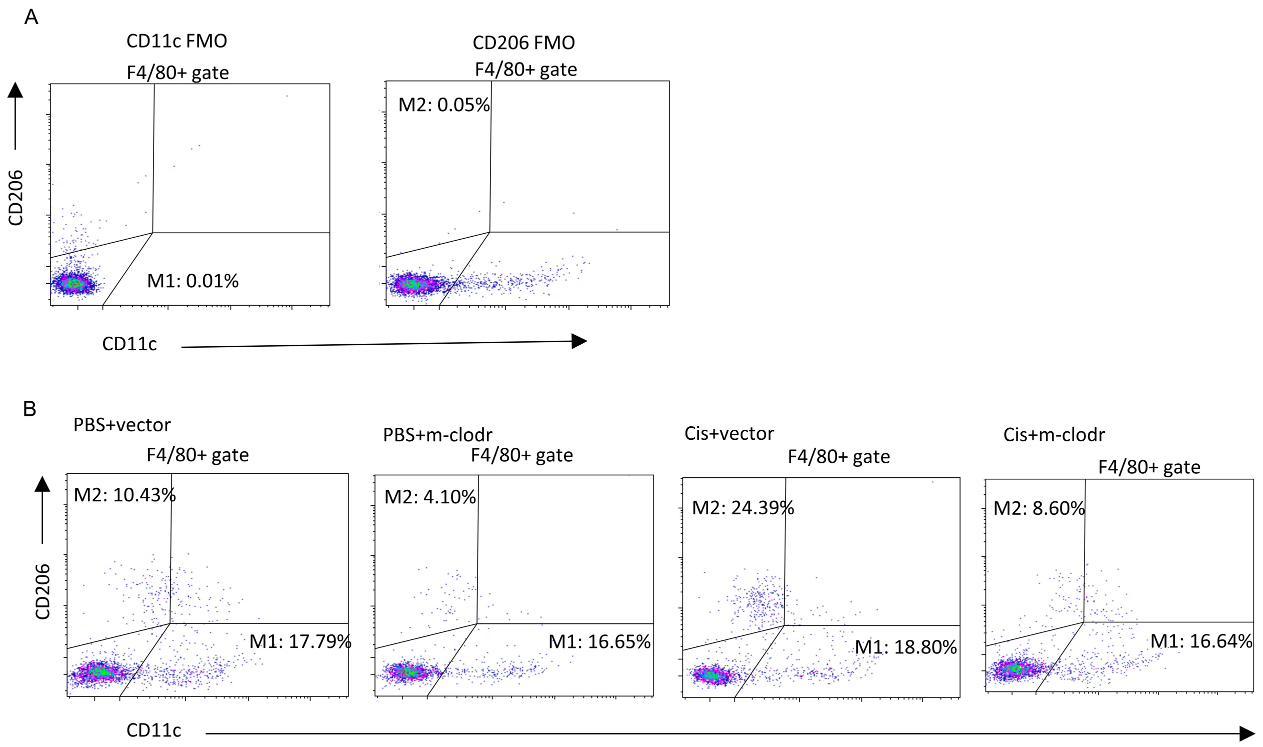

Supplemental Figure 8. Flow cytometry plots for analysis of the effect of mannosylated-clodrosome on DRG macrophages. F4/80+ DRG macrophages were gated as in supplementary figure 2A and analyzed for M2 (CD206+CD11c-) and M1 (CD206-CD11c+) macrophages. **(A)** FMO for this experiment as assessed using splenocytes. **(B)** Representative flow cytometry plots for the data presented in figure 4C and D.

Supplemental Figure 9

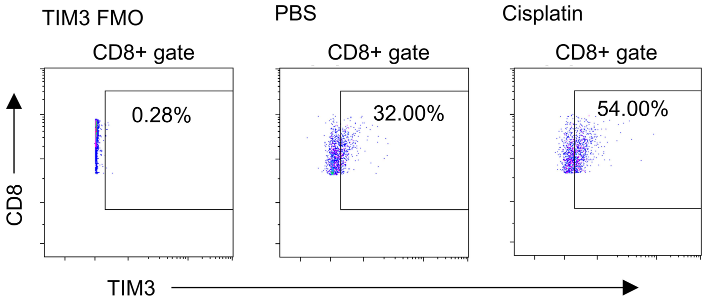

Supplemental Figure 9. Flow cytometry examples of TIM3+CD8 T cells in DRG. TIM3 FMO used for identification of TIM3+CD8 T cells and examples of the TIM3+CD8 T cells in DRG for the data presented in Figure 5A.

Supplemental Figure 10

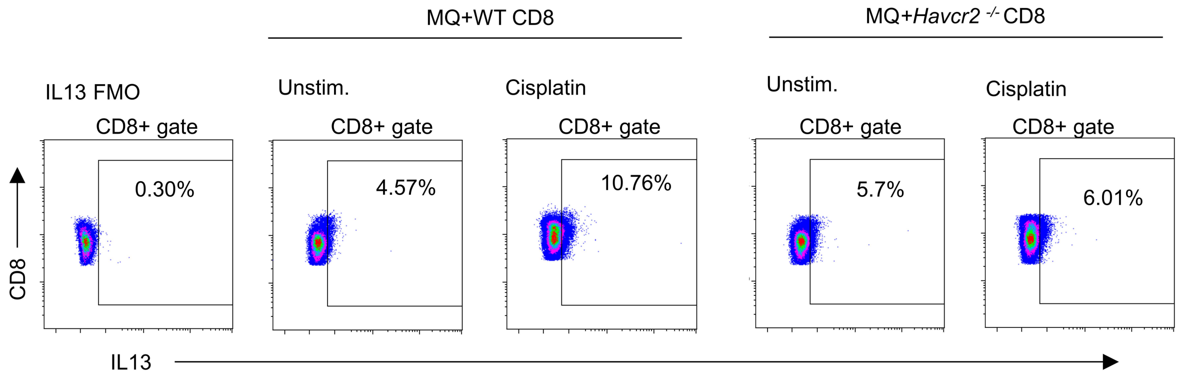

Supplemental Figure 10. Flow cytometry examples of IL13+CD8 T cells in co-cultures of BMDM with CD8 T cells. IL13 FMO used for identification of IL13+ CD8 T cells and examples of the IL13+ CD8 T cells in representative cultures for the data presented in Figure 5B.

Supplemental Figure 11

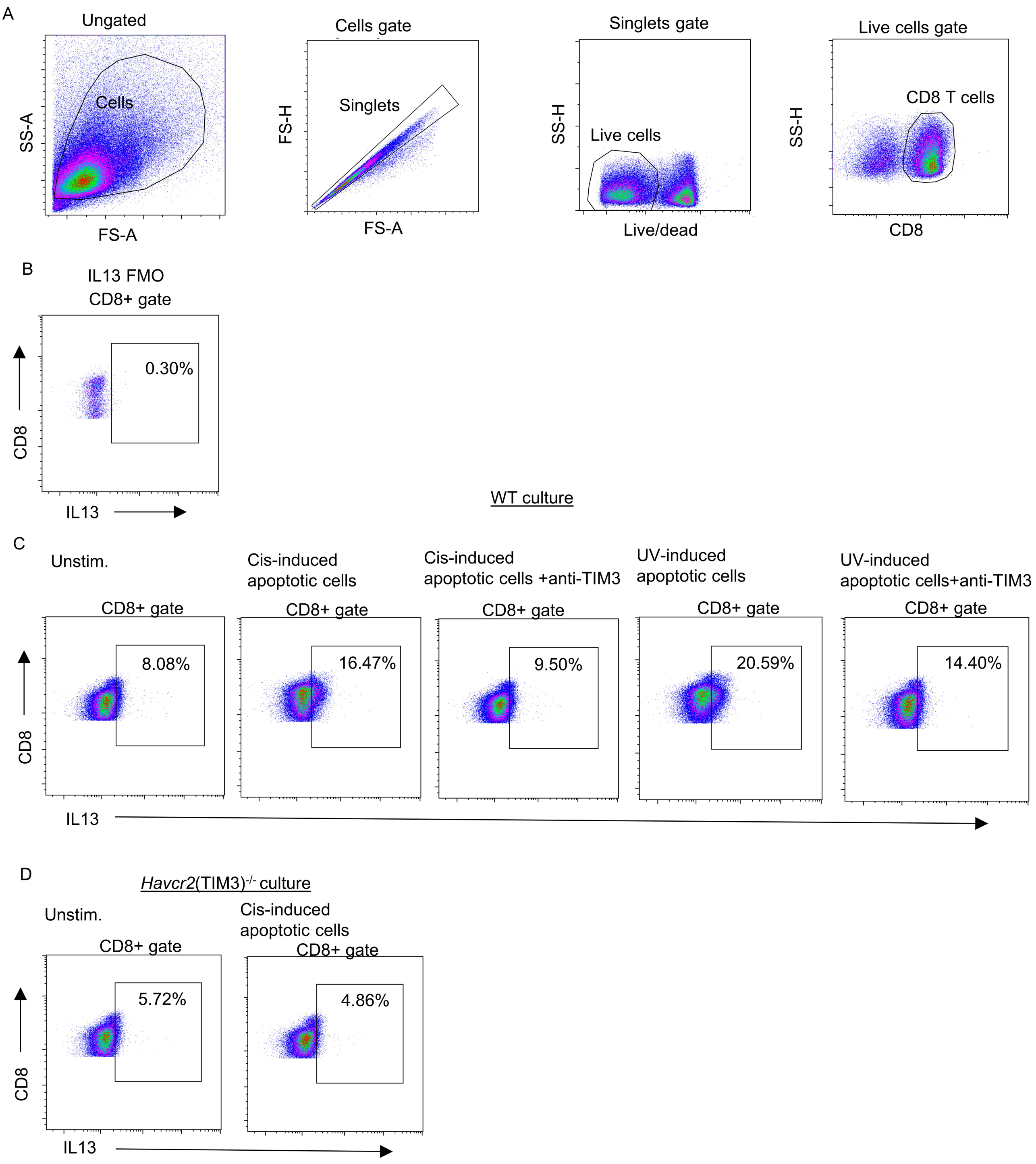

Supplemental Figure 11. Flow cytometry gating and examples of IL13+CD8 T cells in apoptotic cells co-culture with CD8 T cells. (A) Gating strategy: Singlets were selected, followed by gating for CD45+ leukocytes, and live cells followed by selection of CD8 T cells for analysis of IL13+ CD8 T cells. **(B)** IL13 FMO used for identification of IL13+ CD8 T cells and **(C, D)** examples of the IL13+ CD8 T cells in representative cultures of with WT (C; corresponding to Figure 5D) and *Hacr2*(TIM3)$^{-/-}$ cells (D, corresponding to Figure 5E).

Supplemental Figure 12

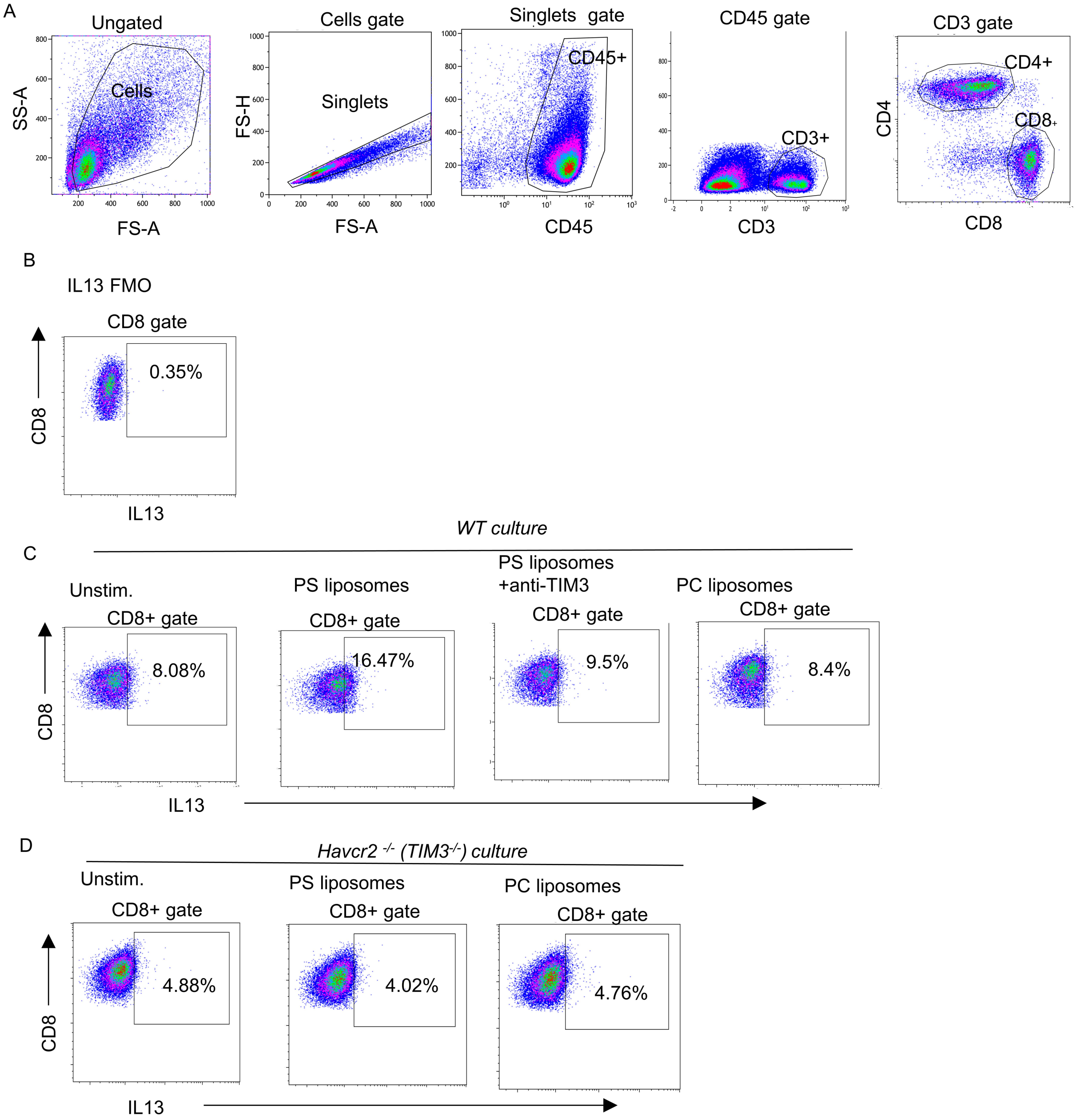

Supplemental Figure 12. Flowcytometry gating strategy of splenocytes and representative example of analysis of IL13+CD8 T cells in cultures stimulated with PS/PC liposomes. A: Gating strategy: Singlets were selected, followed by gating for CD45+ leukocytes and then CD3 T cells, followed by selection of CD8 T cells. **B:** IL13 FMO and **C, D:** Representative examples of IL13+ WT CD8 T cells (C) and *Havcr2*(TIM3)$^{-/-}$ CD8 T cells (D) corresponding to the data presented in Figure 5D-E).

Supplemental Figure 13

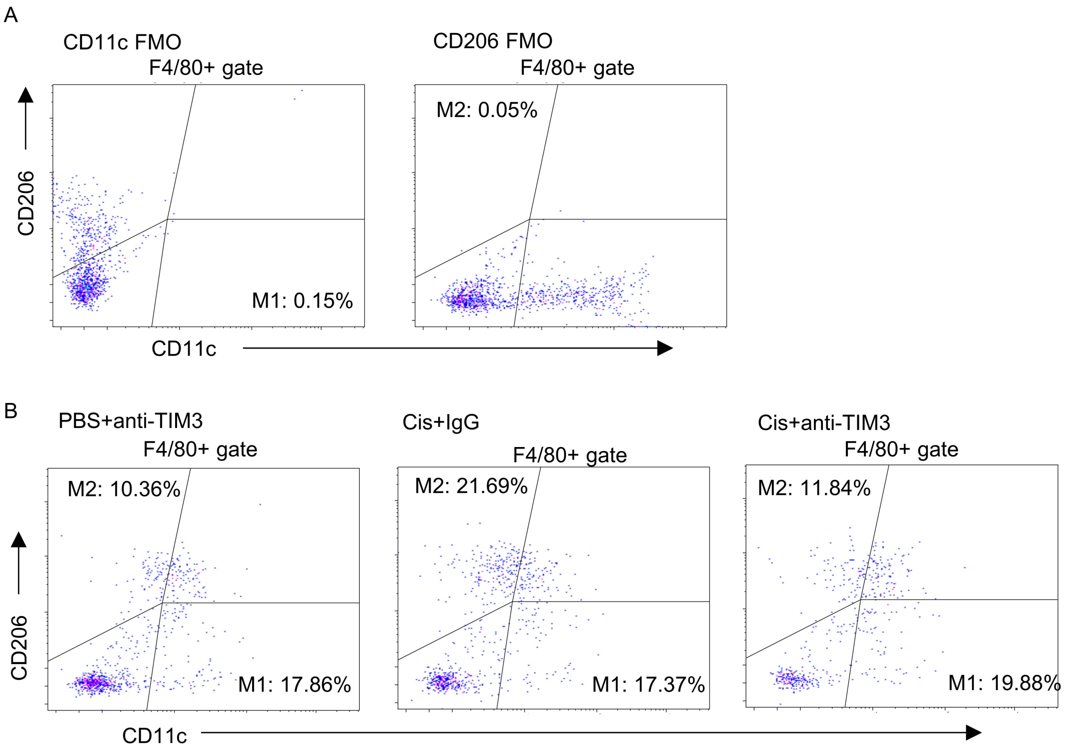

Supplemental Figure 13. **Flow cytometry plots for analysis of the effect of intrathecal anti-TIM3 administration on DRG macrophages.** F4/80+ DRG macrophages were gated as in supplementary figure 2 and analyzed for M2 (CD206+CD11c-) and M1 (CD206-CD11c+) macrophages . **(A)** FMO for this experiment as assessed using splenocytes. **(B)** Representative flow cytometry plots for the data presented in figure 6C and D.

Supplemental Figure 14

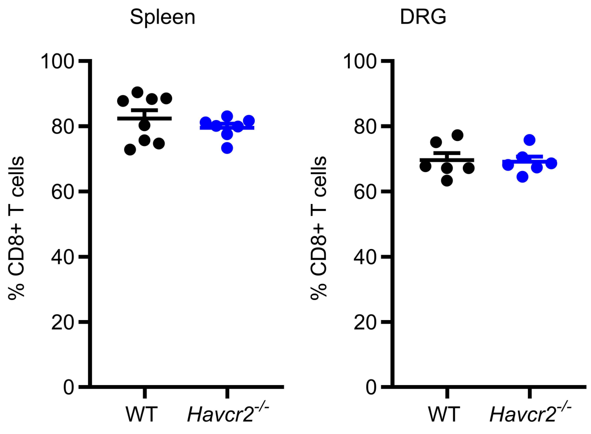

Supplemental Figure 14. Reconstitution of CD8 T cells was similar in *Rag2*$^{-/-}$ mice receiving WT or *Havcr2*$^{-/-}$ CD8 T cells. Flow cytometric analysis of CD8 T cells in the spleen and DRG of *Rag2*$^{-/-}$ mice reconstituted with WT or *Havcr2*(TIM3)$^{-/-}$ CD8 T cells. n= 3-4M+3-4F/group. Welch's T-test: ns.
